# Costs of surgeries in low- and middle-income countries: a systematic literature review

**DOI:** 10.1136/bmjgh-2025-020596

**Published:** 2026-09-04

**Authors:** Martilord Ifeanyichi, Phyllis Tenkorang, Esther Matey, Chizowa Okwuchukwu Ezeaku, Oluwayemisi Ekor, George Duke Mukoro, Soham Bandyopadhyay, Jose Luis Mosso-Lara, Pedra Rabiee, Danyal Zaman Khan, Maeve Bognini, Meskerem Aleka Kebede, Rachel Hargest, Rocco Friebel

**Affiliations:** 1 Global Surgery Policy Unit, LSE Health, The London School of Economics and Political Science, London, UK; 2 African Centre for Health Economics and Policy Research, Enugu, Nigeria; 3 Global Anaesthesia, Surgery and Obstetric Collaboration, Newcastle, UK; 4 Department of General Surgery, Auckland City Hospital, Auckland, New Zealand; 5 Department of Neurosurgery, Obafemi Awolowo University Teaching Hospital Complex, Ife, Osun, Nigeria; 6 Anaesthesia and Pain Management, University of Cape Coast School of Medical Sciences, Cape Coast, Ghana; 7 Department of General Surgery, Ahmadu Bello University Teaching Hospital, Zaria, Kaduna, Nigeria; 8 Nuffield Department of Surgical Sciences, Oxford University Global Surgery Group, University of Oxford, Oxford, UK; 9 Addenbrooke’s Hospital, Department of Obstetrics and Gynaecology, Cambridge University Hospitals NHS Foundation Trust, Cambridge, UK; 10 Queen Square Institute of Neurology, University College London, London, UK; 11 School of Medicine, University Hospital of Wales, Cardiff, Cardiff, UK; 12 Department of Health Policy, The London School of Economics and Political Science, London, UK

**Keywords:** Health economics, Health policy, Public health, Global surgery

## Abstract

**Background:**

Surgical care is essential for achieving global health equity, yet low- and middle-income countries (LMICs) face major gaps in access and planning, partly due to limited evidence on the costs and resource requirements of surgical interventions. Understanding these costs is vital for designing efficient and equitable health systems.

**Methods:**

We conducted a systematic literature review (covering MEDLINE, EMBASE, Global Health, EconLit and grey literature) to identify studies reporting the costs of surgeries in LMICs from January 2000 to June 2023. Minor and major surgical procedures were considered, focusing on therapeutic procedures (excluding diagnostic interventions). Studies that clearly identified, quantified and costed hospital resources and services deployed in the provision of surgical care, and included at least two of the surgical production factors (ie, consumables, diagnostics, personnel, infrastructure and overhead) in the costing were included. Costs were standardised to 2023 International dollars (I$) for comparability.

**Results:**

A total of 74 studies from 29 countries met the inclusion criteria, with 210 cost estimates across 65 procedure groups. Costs varied widely: from I$1.54 for a caesarean section in Tanzania to I$618 098 for paediatric cataract surgery in Zambia. Full costing studies reported higher estimates than partial costing studies. Most studies (60%) originated from upper-middle-income countries, with limited data (10%) from low-income settings.

**Conclusion:**

This review provides a reference list of surgical procedure costs across LMICs, highlighting considerable cost variation by procedure, specialty and country. The findings underscore the need for better-quality, standardised cost data—especially from low-income countries—to inform national surgical plans, universal health coverage benefit packages and reimbursement policies.

**WHAT IS ALREADY KNOWN ON THIS TOPIC**

- While surgical conditions account for a significant portion of the global disease burden, economic evidence—particularly costing data—for surgical services in low- and middle-income countries (LMICs) is sparse. Existing studies are few, often focus on single procedures or contexts, and lack methodological consistency, thus limiting the ability of policymakers and funders to make informed decisions about integrating surgical care into health financing and planning frameworks.

**WHAT THIS STUDY ADDS**

- This systematic review provides the most comprehensive synthesis to date of published cost data on surgical procedures across LMICs, encompassing 210 cost estimates across 65 procedure groups from 29 countries. The study offers a structured reference list of surgical costs for use in economic evaluations, health technology assessments and the development of national surgical and universal health coverage plans.

**HOW THIS STUDY MIGHT AFFECT RESEARCH, PRACTICE OR POLICY**

- Researchers may build on this work to expand cost evidence in low-income and under-represented settings, while implementers and planners can use the findings to optimise resource allocation, integrate cost-effective surgical procedures into benefit packages and pursue cost-reduction strategies such as pooled procurement or task shifting.

## Introduction

 Economic evidence is relied on by policymakers for resource allocation decisions that aim to maximise population benefits from deployed investments. Costing, either as a stand-alone exercise or a precursor of cost-effectiveness analysis, sheds light on resource requirements, resource flows and opportunities for cost reduction in the provision of specific interventions. It therefore facilitates efficiency, fairness and transparency in planning and allocation decisions.^1 2^ A recent review of evidence has shown that economic evaluations in low- and middle-income countries (LMICs) have focused disproportionately on infectious diseases, neglecting other crucial areas, such as chronic and surgical conditions.^3^

There is a substantial unmet need for surgery in LMICs where an estimated one in nine persons lack access to safe, timely and affordable surgical care, causing a major threat to the health and economic development of populations.^4^ A lack of investment in surgery adversely impacts the global aspirations of shared prosperity as represented by the Sustainable Development Goals especially 1, 2 and 3.^5^ A concerted policy effort is required to generate resources^6^ and direct investments for strengthening health systems to deliver surgical care, yet insights from a series of national surgical plans, mostly developed in countries located in sub-Saharan Africa, have highlighted a dearth of evidence on surgical costs and resource requirements with likely implications for effective planning and policy implementation.^7^ This evidence gap may be driven by the heterogeneity in surgical procedures, which range from minor interventions (eg, lumpectomy) to highly specialised interventions (eg, cardiac transplants), aside that the costing of surgeries is resource-intensive and technically challenging.^8^

Commonly, there are three steps in costing healthcare services: (1) identifying all the resources used in delivering the service, including ideally both direct and indirect (overhead) costs; (2) quantifying the resources consumed in natural units; and (3) attaching monetary values to the estimated resource use.^1^ Information can be quantified either in a bottom-up approach (micro-costing, using activity and resource data derived from direct observations, for instance), or a top-down approach (macro-costing, using overall expenditures for each input variable at a central level and then allocating these to specific procedures using selected allocation factors such as building usage, staff time and staff numbers). In simple terms, bottom-up costing estimates costs by identifying and summing the costs of each individual resource or activity used in delivering a service while top-down costing starts with total expenditure at an aggregate level and allocates costs across services or activities using allocation rules or averages. However, the choice of approach remains contested.^9^

The existing studies that provide costing information are often limited to a few selected surgical procedures and narrow geographical settings.^10 11^ Our study aims to develop a reference list of costs associated with surgical procedures by systematically compiling and characterising the available evidence from a wide range of health systems, surgical specialities and surgical procedures. Information will then be available for use by policymakers across LMICs to inform evidence-based policy reforms aimed at strengthening local surgical systems (eg, the adaptation of national surgical plans). Moreover, costing data may also be relevant for the refinement of the Universal Health Coverage Compendium, provided to member states to develop needs-based benefit packages by the WHO.^12^

## Methods

We conducted a systematic literature review to synthesise the evidence on the cost of surgeries in LMICs in line with the guidelines published by the Centre for Reviews and Dissemination (CRD),^13^ and the Cochrane Handbook for Systematic Reviews.^14^

### Search strategy and information sources

Our search strategy was based on three search blocks—‘surgery and anaesthesia’, ‘cost’ and ‘low- and middle-income countries’. A detailed search strategy was designed for Ovid MEDLINE and then adapted to EMBASE, Global Health and EconLit (see online supplemental material 1 for full search strategies). All four databases were searched without any language restrictions and for literature published from 01 January 2000 to 01 June 2023. References of identified publications were hand searched for any additional relevant publications, as well as websites of the WHO and World Bank were screened. Conference presentations, abstracts, dissertations and animal studies were excluded.

### Selection procedure

Articles were deduplicated in EndNote and transferred to Rayyan—a web and mobile app designed for systematic reviews^15^—for title and abstract screening. Each article was reviewed in parallel by two researchers independently, with conflicts resolved by discussion and/or arbitration by a senior researcher.

Inclusion and exclusion of articles followed a prespecified set of eligibility criteria, based on an adaptation of the Population, Intervention, Comparator, Outcome and Study Design framework of the CRD.^13^ Minor and major surgical procedures were considered, with a focus on therapeutic surgeries (eg, appendectomy or herniorrhaphy) and excluding diagnostic surgical procedures (eg, biopsy). We included minimally invasive intraluminal and endovascular procedures, whereas extracorporeal treatments like radiotherapy, and neoadjuvant and adjunct interventions were excluded. We included studies conducting pure cost analyses of surgical interventions in LMICs, while economic evaluations (cost-effectiveness analysis, cost-utility analyses, cost-benefit analysis and cost-minimisation analyses) were excluded.^16^ Both full and partial costing studies were included, and we limited our inclusion to studies with clearly described cost components captured. Studies that identified, quantified and costed hospital resources and services deployed in the provision of surgical care were included, while those that only aggregated data from health systems/insurance claims and reimbursement records were excluded. Further, for inclusion, articles must include at least two of the surgical production factors (ie, consumables, diagnostics, personnel, infrastructure and overhead) in the costing. We excluded costing studies that encompassed any patient costs, such as out-of-pocket medical expenses, transportation and productivity losses. The complete list of eligibility criteria is provided in online supplemental material 2.

### Data extraction and quality assessment

A customised form was designed in Microsoft Excel to conduct the data extraction by one researcher, with a second researcher checking for completeness and accuracy of the data. The quality of included articles was assessed using an adjusted version of the 2022 Consolidated Health Economic Evaluation Reporting Standards (CHEERS) checklist.^17^

### Data analysis and reporting

We conducted a descriptive analysis of the evidence on unit costs of production of surgical interventions. In studies that presented only study population total costs, unit costs were computed by dividing the total costs by the number of study subjects or procedures. To allow for comparison of costs from different years, the costs were converted to 2023 equivalents using gross domestic product (GDP) deflators. The inflation-adjusted values were then converted to International dollars (I$) using purchasing power parities to facilitate cross-country comparisons. Due to wide variations in interventions studied, population characteristics (eg, medical condition and severity, gender, age and comorbidities) and methodological approaches (eg, top-down vs bottom-up costing), we provided a narrative synthesis of our findings.^18^ We aggregated surgical interventions by country of study, specialty and procedure group. For example, total abdominal hysterectomies, vaginal hysterectomies, subtotal abdominal hysterectomies and laparoscopic hysterectomies were grouped into ‘hysterectomies’. Medians and ranges were computed and presented. Subgroup analyses were conducted per type of study (full vs partial costing).

The study is registered on the international prospective register for systematic reviews (PROSPERO) under CRD42023432084.

### Patient and public involvement

Patients or the public were not involved in the design, conduct, or reporting, or dissemination plans of our research.

## Results

**Figure 1:**
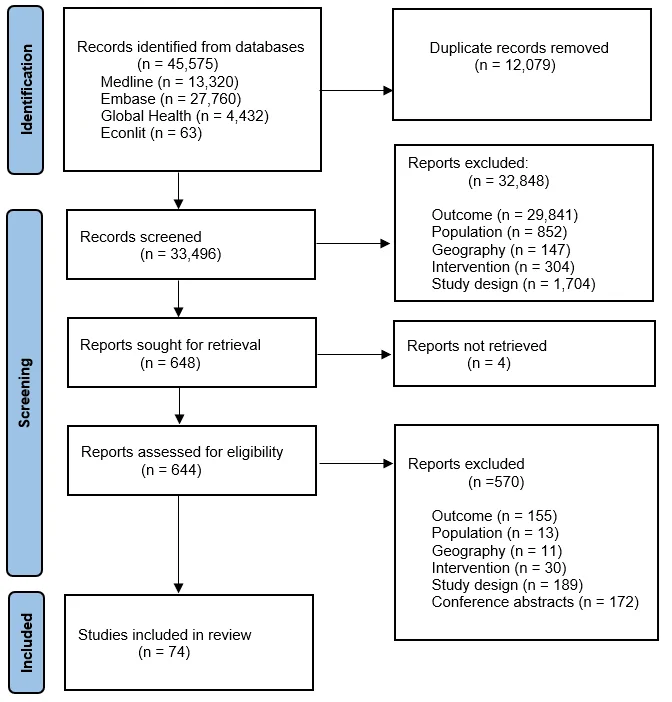
PRISMA flow chart. PRISMA, Preferred Reporting Items for Systematic Reviews and Meta-Analyses.

The search identified a total of 45 575 articles, which yielded 33 496 unique articles following deduplication. A total of 648 articles were full-text reviewed, with 74 articles meeting the inclusion criteria for the review (see figure 1).

The identified studies spanned 29 countries, predominantly from Brazil (n=13), India (n=10) and China (n=10), which contributed close to half of the studies (44%). A total of 45 studies (60%) were from upper-middle-income countries, while less than 10% (n=7) provided evidence from low-income countries. Overall, 18 studies focused on sub-Saharan Africa (24%), 17 studies on Latin America and the Caribbean (23%) and 16 studies on East Asia and Pacific (22%). A total of 12 specialties were covered in the review, with most evidence relating to obstetrics and gynaecology (n=13), transplant surgery (n=12) and orthopaedic surgery (n=10). An overview of all included studies and their characteristics is provided in online supplemental material 3.

### Costing methodologies

A total of 44 studies (59%) took a full costing approach (ie, including elements of the overhead costs), and 30 studies (41%) conducted a partial costing study (ie, including only the direct clinical care costs). Resource quantification and costing was mostly done using bottom-up costing (n=48 (65%)), while 7 (9%) conducted top-down costing; a quarter of the studies (n=19) combined top-down and bottom-up methods. Resource use and cost data were collected mostly retrospectively (n=55) or prospectively (n=16). Among the full costing studies, 18 studies applied micro-costing, 7 studies adopted macro-costing and 19 studies combined micro-costing and macro-costing methods. In most of the full costing studies, direct medical costing involved explicit definition and quantification of resource use through, for instance, interviews, (time-driven) activity-based costing or time and motion approaches. Unit prices were drawn from various sources, including hospital procurement records (n=13), hospital billing records (n=8) or hospital reference prices (n=7). All the (30) partial costing studies conducted micro-costing and involved a review of actual individual patient care utilisation and associated bills, as recorded in the hospital medical and billing records. Detailed information about the underlying costing methodologies is presented in online supplemental material 3.

### Costs of surgeries

We identified 210 cost estimates across 65 procedure groups. Across all the 210 cost points, the cheapest procedure was caesarean section conducted in Tanzania at the cost of I$1.54, while the most expensive procedure was paediatric cataract surgery in Zambia at I$618 098. Across all procedure groups, the cheapest interventions were surgical treatment of genital warts (median cost I$42) and the most costly being refractive error surgeries (median cost I$164 948.50). Across all countries, the median costs of all surgeries ranged from I$42 in Peru to I$22 566.91 in Nigeria. Across all specialties, the median costs ranged from I$113.60 for paediatric surgery to I$32 979.54 for transplant surgery. Information about medians and ranges of costs of surgeries across all the identified intervention clusters, countries and specialties is presented in tables 1–3.

**Table 1:** Cost summaries by procedure groups

S/N	Procedure groups	Number of cost data points	Median (I$)	Minimum (I$)	Maximum (I$)
1	Genital warts treatment	3	42.00	37.09	59.22
2	Capsulotomy	1	94.78	94.78	94.78
3	Male circumcision	1	113.60	113.60	113.60
4	Cystoscopy	1	164.58	164.58	164.58
5	Electrosurgery	1	247.26	247.26	247.26
6	Treatment of atonic PPH	1	257.81	257.81	257.81
7	Cutaneous swellings excision	1	313.19	313.19	313.19
8	Skin carcinoma treatment	1	411.93	411.93	411.93
9	Lithotomy	3	428.22	239.80	861.29
10	Salpingectomy	2	515.45	341.14	689.77
11	Fasciectomy	1	527.48	527.48	527.48
12	Intestinal obstruction surgery	1	538.61	538.61	538.61
13	Injury surgical treatment	8	570.42	12.05	8744.19
14	Pterygium surgery	1	576.93	576.93	576.93
15	Hydrocelectomy	1	607.30	607.30	607.30
16	Glaucoma surgery	1	704.68	704.68	704.68
17	Septoplasty	1	745.89	745.89	745.89
18	Caesarean section	28	794.68	1.54	4785.41
19	Tympanoplasty	1	803.58	803.58	803.58
20	Adenoidectomy	2	911.97	313.19	1510.75
21	Haemorrhoidectomy	1	935.45	935.45	935.45
22	Appendectomy	8	972.03	203.55	25 099.15
23	Prostatectomy	1	980.91	980.91	980.91
24	Arthrodesis	1	1083.81	1083.81	1083.81
25	Hernia repair	9	1110.17	189.35	4906.46
26	Sinus surgery	3	1203.31	684.08	4489.62
27	Hysterectomy	10	1205.88	346.74	4844.76
28	Thyroidectomy	1	1244.52	1244.52	1244.52
29	Adenectomy	1	1330.81	1330.81	1330.81
30	Tonsillectomy	1	1330.81	1330.81	1330.81
31	Cranioplasty	2	1400.04	1278.50	1521.57
32	Long bone fracture fixation	2	1511.75	706.76	2316.73
33	Spine surgery	1	1541.10	1541.10	1541.10
34	Mastectomy	1	1557.72	1557.72	1557.72
35	Myomectomy	3	1559.48	1334.56	1889.37
36	Hydrocephalus surgery	2	1696.87	1102.44	2291.30
37	Myelomeningocele repair	1	1718.36	1718.36	1718.36
38	Cataract surgery	14	1783.77	247.26	618 098.20
39	Orthopaedic tumour surgery	1	2126.45	2126.45	2126.45
40	Craniotomy	11	2195.31	1098.56	11 694.33
41	Cholecystectomy	6	2289.27	1244.78	3711.39
42	Cardiac defibrillator implantation	1	2910.03	2910.03	2910.03
43	Limb deformities procedures	1	3267.96	3267.96	3267.96
44	Removal of orthopaedic hardware	1	3376.48	3376.48	3376.48
45	Laparotomy	3	3662.36	1893.12	4417.63
46	Ankle and foot procedures	1	3673.88	3673.88	3673.88
47	Elbow, shoulder and knee repairs	1	3947.40	3947.40	3947.40
48	Surgical debridement	1	4412.29	4412.29	4412.29
49	Hepatectomy	4	4776.90	4469.00	5759.00
50	Cardiac valve repair	6	5562.15	563.02	39 672.96
51	Oral and maxillofacial reconstruction	3	5732.90	1046.36	5921.19
52	Amputation	2	6457.57	433.68	12 481.47
53	Bariatric surgery	2	7176.93	6837.09	7516.77
54	Hip fracture repair	11	7383.92	352.35	18 333.76
55	Catheter ablation	1	7930.27	7930.27	7930.27
56	Knee arthroplasty	5	11 093.14	6536.30	21 084.43
57	Cardiac revascularisation	8	12 273.27	2495.15	22 566.91
58	Orthopaedic infection treatment	2	12 759.32	4024.10	21 494.53
59	Kidney transplant	4	13 115.66	6573.55	20 988.00
60	Congenital heart surgery	2	17 778.44	16 677.56	18 879.31
61	Simultaneous pancreas-kidney transplantation	2	37 306.14	41 632.74	32 979.54
62	Liver transplant	3	38 573.83	37 196.28	76 326.43
63	Bile duct injury repair	4	49 965.34	49 965.34	49 965.34
64	Heart transplant	1	50 328.38	644.41	167 393.60
65	Refractive error surgery	3	164 948.50	164 948.50	164 948.50

I$, International dollars; PPH, postpartum haemorrhage.

**Table 2:** Cost summaries by specialty

S/N	Specialty	Number of cost data points	Median (I$)	Minimum (I$)	Maximum (I$)
1	Paediatric surgery	1	113.60	113.60	113.60
2	Urology	6	517.76	164.58	980.91
3	Obstetrics and gynaecology	48	830.48	1.54	4844.76
4	Ear, nose and throat	9	1203.31	313.19	4489.62
5	Ophthalmology	18	1266.75	94.78	618 098.20
6	Neurosurgery	17	1891.14	1098.56	11 694.33
7	General surgery	39	2246.88	189.35	49 965.34
8	Plastic surgery	4	3389.63	411.93	5921.19
9	Orthopaedic surgery	37	4009.14	12.05	21 494.53
10	Interventional cardiology	7	7930.27	2495.15	19 161.14
11	Cardiothoracic surgery	11	13 839.00	563.02	39 672.96
12	Transplant surgery	13	32 979.54	644.41	167 393.60

I$, International dollars.

**Table 3:** Cost summaries by country

S/N	Country	Number of cost data points	Median (I$)	Minimum (I$)	Maximum (I$)
1	Peru	3	42.00	37.09	59.22
2	Malawi	10	292.47	189.35	881.80
3	Sri Lanka	5	428.22	164.58	980.91
4	Indonesia	1	566.98	566.98	566.98
5	Iran, Islamic Republic	6	621.75	480.77	6573.55
6	Ethiopia	1	688.53	688.53	688.53
7	Bangladesh	2	824.09	716.09	932.08
8	Morrocco	1	880.96	880.96	880.96
9	India	56	949.88	12.05	84 672.98
10	Sudan	1	1046.36	1046.36	1046.36
11	Tanzania	22	1072.14	1.54	4412.29
12	Uganda	13	1718.36	1098.56	4600.75
13	Cambodia	2	1892.12	1542.33	2241.91
14	Rwanda	2	2408.53	1154.69	3662.36
15	Malaysia	3	2528.32	2127.64	10 707.53
16	Mongolia	1	2933.09	2933.09	2933.09
17	Kenya	9	3947.40	2126.45	11 093.14
18	Zambia	7	4906.46	3071.92	618 098.20
19	China	19	5052.80	352.35	39 672.96
20	Turkey	10	5122.12	1244.78	76 326.43
21	Mexico	5	7383.92	4009.14	19 161.14
22	South Africa	16	8744.19	113.60	49 965.34
23	Brazil	2	8781.78	411.93	167 393.60
24	Serbia	1	11 276.34	8140.68	14 412.01
25	Egypt	1	11 694.33	11 694.33	11 694.33
26	Jordan	2	12 481.47	12 481.47	12 481.47
27	Palestine	3	12 547.75	5921.19	19 174.31
28	Papua New Guinea	3	21 621.39	21 249.97	25 099.15
29	Nigeria	3	22 566.91	16 677.56	29 982.13

I$, International dollars.

In the subgroup analyses, we identified a total of 150 cost estimates across 49 procedure groups among the full costing studies, and a total of 29 procedure groups with 60 cost data points across the partial costing studies. Costs were generally higher in the full costing studies. Across the full studies, procedure groups, median costs ranged from I$42 for surgical treatment of genital warts to I$164 948.50 for refractive error surgeries. Among the partial studies, the median costs ranged from I$411.93 for skin carcinoma surgeries, while the most expensive were costing I$76 326.43 for transplant surgeries. Further results from the subgroup analyses linked to full or partial costing estimates are presented in online supplemental materials 4 and 5, respectively.

### Quality assessment of included studies

We identified significant heterogeneities in the overall quality of included studies, with wide variation in adherence to specific items of the CHEERS checklist. While almost all studies (96%) provided context for the study, the research question, its practical relevance for decision-making in policy or practice and most described the intervention being costed and reason for its choice (88%), fewer than 65% of studies reported the dates of the estimated resource quantities and unit costs, the currency and year of conversion. Moreover, 32% of studies described how uncertainty about analytical judgements, inputs or projections affected findings (see full overview of adherence to checklist items across all studies in online supplemental material 6).

## Discussion

Evidence of costs and resource requirements is important for decision-making and resource allocation. By synthesising cost estimates provided in 74 studies, spanning 29 countries, we present important reference data for 65 surgical procedure groups and 12 surgical specialties. We found large variation in costs across procedures, ranging from I$1.54 for caesarean section in Tanzania to I$618 098 for paediatric cataract surgery in Zambia, and within specialties, ranging from I$4844.76 to I$1.54 for obstetrics and gynaecology. Notably, most studies originated from upper middle-income countries, with limited data from low-income countries. By distinguishing between full and partial costing methodologies, we show that cost estimates tend to be higher for full costing studies compared with partial costing approaches. This emphasises the importance of adopting comprehensive costing approaches to derive more meaningful estimates for integration into local resourcing decisions.

Surgeries are often perceived as expensive, especially when compared with traditional public health interventions (eg, tuberculosis treatment, HIV prevention, breastfeeding campaigns, salt iodisation and childhood immunisation) that have received substantial local and international funding over the past decades. Based on our findings, surgeries are indeed expensive. For example, pre-natal micronutrient supplementation costs US$2.60 throughout a woman’s pregnancy^19^; the production of a new two times per year shots for HIV prophylaxis (lenacapavir) is projected to cost US$40 per treatment under generic production^20^; and the per person cost of distributing insecticide treated nets ranges from US$0.88 to US$9.54.^21^ Costs for surgical procedures widely exceed the cost of such interventions.

The high cost of surgical procedures is likely a consequence of its complexity and horizontal integration into the whole healthcare system. Cost drivers include the costs associated with well-equipped surgical theatres and wards, trained personnel (surgeons, anaesthetists, nurses and support staff), medical and blood supplies, administration of financing mechanisms and governance structures. As Farmer and Kim succinctly put it, "there is no surgical equivalent to a vaccination campaign or a mosquito net”.^22^

However, a growing body of evidence has shown that when considering the cost per unit of health gain, low- to moderate-complexity surgical procedures compare favourably to, and in some instances are more cost-effective than conventional public health interventions.^16 23^ Moreover, the opportunity cost to society at large of not providing surgery is potentially enormous. In 2015, the Lancet Commission on Global Surgery estimated that scaling up surgical services in LMICs would require an investment of US$350 billion to US$420 billion by 2030. Failing to mobilise investments into surgical care, on the other hand, was estimated at a US$12.3 trillion loss in GDP due to lost productivity. More so, using internal instruments techniques to address endogeneity, a recent study has demonstrated a statistically significant positive association between surgical activity and economic development in LMICs, suggesting that surgery may be not just a health intervention but a driver of economic prosperity.^24^

Despite the growing recognition of surgical care as an essential component of global health, robust evidence on the costs of surgical procedures remains limited.^4^ We identified only 74 costing studies over 23 years, with only half of those studies providing comprehensive (full) costing estimates. This is particularly inadequate given the vast range of surgical procedures available, each with varying levels of complexity, resource requirements and settings of delivery. Moreover, the existing studies were predominantly from upper-middle-income countries, with virtually no or limited representation from low-income countries and African nations, where the burden of surgical disease is highest. This evidence gap is in line with previous findings in other reviews,^16 25^ which carries direct implications for policymakers in low-resource settings when determining surgical benefit packages that are tailored to local population needs and inclusive of costing implications. Future research must strive for the provision of detailed, clinically and geographically diverse costing estimates grounded in methodological transparency and consistency in reporting to enhance comparability and usability of such data for policy and planning.

### Strengths and limitations

Our study has several strengths. We adhered to the Cochrane Handbook of Systematic Reviews, CRD Guidance and Preferred Reporting Items for Systematic Reviews and Meta-Analyses (PRISMA), ensuring reliability and replicability. The inclusion of full and partial costing studies enhanced the depth of analysis, allowing for a nuanced interpretation of cost variations. However, there are some limitations. First, the heterogeneity across studies regarding costing methodologies, reporting practices and study populations precluded the use of a meta-analysis to synthesise findings. However, presenting the costs as medians and ranges allowed the aggregation of costs of otherwise disparate interventions across different procedure groups, countries and specialties. Second, the limited breadth of procedures covered in this review limits the extent to which our comparisons especially across countries and specialties represent the real-world variations in surgical costs. For instance, our study finds that in Peru, surgeries cost between I$37 and I$60 whereas in Nigeria they cost I$16 678 to I$29 982. This is more likely an artefact of which surgical procedures were included in the articles included in the review rather than a true difference in the costing of surgery between these two countries. Third, the reliance on retrospective data in most studies introduces potential biases related to data completeness and accuracy. Only a small proportion of costing data were generated using prospective collection methods. Fourth, the concentration of studies in upper-middle-income countries limits generalisability to low-income settings, where cost structures may differ substantially. Fifth, the exclusion of non-medical patient costs (eg, transportation and lost productivity) means that the full societal costs of surgical care are not fully captured in our estimates. Lastly, this study fails to capture the heterogeneities in the ownership (private, public, faith-based, non-governmental organisations), funding mechanisms (government, patient, donors), financial goals (profit vs non-profit) and settings (urban vs rural) of surgical service providers in LMICs as the relevant disaggregated analyses were not included. Addressing these limitations in future research through standardised costing frameworks, inclusion of broader cost components and context-appropriate disaggregated analyses will be essential for strengthening the evidence base for surgical cost analyses in LMICs.

### Policy implications and conclusion

The findings of this review have the potential to serve as reference data, and they hold several policy implications for national surgical planning and health service financing. Robust cost data are essential for priority-setting decisions, ensuring that surgical interventions are appropriately integrated into essential health benefit packages to achieve universal health coverage by 2030. Moreover, national surgical plans must account for the full economic costs of procedures to ensure adequate resource allocation, workforce planning and infrastructural investments. Failure in doing so may be a contributory factor for the lack of implementation of surgical plans observed across LMICs.^26^ In addition, governments, payers including insurance providers, non-governmental organisations, international organisations and funders may use these cost estimates for calibrating reimbursement schemes and to prevent underfunding of surgical services, which could compromise quality and access to care. Our findings provide insights into cost variations across settings that could help inform regional and national strategies for cost reduction, such as pooled procurement of surgical supplies, task-shifting to optimise human resources and investments in cost-effective surgical innovations.

Operative surgical conditions are associated with nearly 18 million deaths and 620 million disability-adjusted life years globally every year.^27^ Concerted efforts are required to develop strategies that address this growing surgical need, including those that address resource allocation and wider planning decisions. Our study fills an important gap in the global surgery economics literature by providing a comprehensive reference list of costs associated with 65 surgical procedure groups. Yet, there remains, however, overall, a paucity of cost evidence, particularly among low-income countries and countries in sub-Saharan Africa. Future research should prioritise data collection in these settings, adopt standardised costing methodologies and explore innovative strategies to optimise surgical costs while maintaining high-quality care.

## Supplementary material

10.1136/bmjgh-2025-020596 online supplemental file 1

10.1136/bmjgh-2025-020596 online supplemental file 2

10.1136/bmjgh-2025-020596 online supplemental file 3

10.1136/bmjgh-2025-020596 online supplemental file 4

10.1136/bmjgh-2025-020596 online supplemental file 5

10.1136/bmjgh-2025-020596 online supplemental file 6

10.1136/bmjgh-2025-020596 online supplemental file 7

## Data Availability

Data are available upon reasonable request.
