# Supplementary material for: Costs of surgeries in low- and middle-income countries: a systematic literature review
Source: BMJ Glob Health. 2026 Sep 4;11(9):e020596. doi: 10.1136/bmjgh-2025-020596 (PMC13548284; doi:10.1136/bmjgh-2025-020596)
Supplement: Supplementary file 2 [file bmjgh-11-9-s002.pdf]

Supplemental material 2: Inclusion and exclusion criteria

PICOS	Inclusion	Exclusion
Population	• Surgery<br>• Anaesthesia<br>• Obstetric<br>• Surgical gynaecological	• Non-surgery<br>• Medical obstetric<br>• Medical gynaecological
Intervention	• Major surgeries<br>• Minor surgeries	• Diagnostics<br>• Medical<br>• Screening
Comparator	• Not applicable	• Nor applicable
Outcomes	• Provider costs	• Clinical effectiveness<br>• Cost-effectiveness<br>• Resource use (alone)<br>• Patient/household costs
Setting	• Hospitals<br>• Health centres<br>• Primary healthcare facilities<br>• Surgical camps/outreaches<br>• Low- and middle-income countries	• High-income country settings
Study design	• Bottom-up/Micro-costing<br>• Step-down/macro-costing<br>• Mixed methods	• Cost minimisation analyses<br>• Cost comparison analysis<br>• Animal studies<br>• Opinions/editorials/commentaries
Language	• Not applicable	• Not applicable
Time Limit	• From 2000	• Pre-2000
