# Supplementary material for: Costs of surgeries in low- and middle-income countries: a systematic literature review
Source: BMJ Glob Health. 2026 Sep 4;11(9):e020596. doi: 10.1136/bmjgh-2025-020596 (PMC13548284; doi:10.1136/bmjgh-2025-020596)
Supplement: Supplementary file 3 [file bmjgh-11-9-s003.pdf]

Supplemental material 3: Overview of included studies and their results

S/N	Author last name, year	Country	Income group	Specialty	Broad intervention group	Specific intervention description	Costing methodology	Costing type Full vs partial	Reference price	Data collection Retrospective vs prospective	Perspective	Unit costs in 2023 I$
1	Abdelgadir, 2017[1]	Uganda	LIC	Neurosurgery	Craniotomy	Burr holes for subdural hematoma	Bottom up	Full	mixed	Retrospective	Hospital	1,098.56
2		Uganda	LIC	Neurosurgery	Craniotomy	Chiari decompression	Bottom up	Full	mixed	Retrospective	Hospital	1,891.14
3		Uganda	LIC	Neurosurgery	Cranioplasty	Cranioplasty	Bottom up	Full	mixed	Retrospective	Hospital	1,521.57
4		Uganda	LIC	Neurosurgery	Craniotomy	Craniotomy/craniectomy +/- duraplasty	Bottom up	Full	mixed	Retrospective	Hospital	1,663.48
5		Uganda	LIC	Neurosurgery	Cranioplasty	Elevation of skull fracture	Bottom up	Full	mixed	Retrospective	Hospital	1,278.50
6		Uganda	LIC	Neurosurgery	Craniotomy	Excision of brain tumour	Bottom up	Full	mixed	Retrospective	Hospital	4,600.75
7		Uganda	LIC	Neurosurgery	Craniotomy	Subdural hematoma/epidural hematoma evacuation	Bottom up	Full	mixed	Retrospective	Hospital	1,191.05
8		Uganda	LIC	Neurosurgery	Craniotomy	Incision and debridement	Bottom up	Full	mixed	Retrospective	Hospital	2,195.31
9		Uganda	LIC	Neurosurgery	Myelomeningocele repair	Myelomeningocele repair	Bottom up	Full	mixed	Retrospective	Hospital	1,718.36
10		Uganda	LIC	Neurosurgery	Craniotomy	Other neurosurgical procedures	Bottom up	Full	mixed	Retrospective	Hospital	2,174.99
11		Uganda	LIC	Neurosurgery	Craniotomy	Removal of brain abscess or lesion	Bottom up	Full	mixed	Retrospective	Hospital	3,368.38
12		Uganda	LIC	Neurosurgery	Spine surgery	Spine surgery	Bottom up	Full	mixed	Retrospective	Hospital	1,541.10

S/N	Author last name, year	Country	Income group	Specialty	Broad intervention group	Specific intervention description	Costing methodology	Costing type Full vs partial	Reference price	Data collection Retrospective vs prospective	Perspective	Unit costs in 2023 I$
13		Uganda	LIC	Neurosurgery	Craniotomy	Ventriculoperitoneal shunting	Bottom up	Full	mixed	Retrospective	Hospital	2,321.20
14	Abeygunasekera, 2008[2]	Sri Lanka	LMIC	Urology	Cystoscopy	Cystoscopy	Mixed	Full	mixed	Prospective	Hospital	164.58
15		Sri Lanka	LMIC	Urology	Lithotomy	Optical urethrotomy	Mixed	Full	mixed	Prospective	Hospital	239.80
16		Sri Lanka	LMIC	Urology	Lithotomy	Pyelolithotomy	Mixed	Full	mixed	Prospective	Hospital	861.29
17		Sri Lanka	LMIC	Urology	Prostatectomy	Transurethral resection of the prostate	Mixed	Full	mixed	Prospective	Hospital	980.91
18		Sri Lanka	LMIC	Urology	Lithotomy	Vesicolithotomy	Mixed	Full	mixed	Prospective	Hospital	428.22
19	Ahmadi, 2021[3]	Iran, Islamic Rep.	UMIC	Transplant Surgery	Heart transplant	Heart transplant	Bottom up	Full	Hospital billing system	Retrospective	Hospital	644.41
20	Akarsu, 2012[4]	Turkiye	UMIC	Transplant Surgery	Liver transplant	Liver transplant	Bottom up	Partial	Hospital billing system	Retrospective	Hospital	76,326.43
21	Aljarrah, 2020[5]	Jordan	LMIC	General Surgery	Amputation	Non-traumatic major lower extremity amputation	Bottom up	Partial	Hospital billing system	Retrospective	Hospital	12,481.47
22	Allard, 2005[6]	South Africa	UMIC	General Surgery	Laparotomy	Abdominal firearm-related injury surgery	Bottom up	Partial	National reference price	Prospective	Hospital	4,417.63
23	Balakrishnan, 2020[7]	India	LMIC	Transplant Surgery	Heart transplant	Heart transplant	Mixed	Full	Hospital reference prices	Retrospective	Hospital	84,672.98
24	Barreto, 2020b[8]	Brazil	UMIC	Transplant Surgery	Heart transplant	Heart transplant	Bottom up	Partial	Hospital billing system	Retrospective	Hospital	15,983.77
25	Barreto, 2020a[9]	Brazil	UMIC	Transplant Surgery	Kidney transplant	Kidney transplant	Bottom up	Partial	Hospital billing system	Retrospective	Hospital	7,057.02

S/N	Author last name, year	Country	Income group	Specialty	Broad intervention group	Specific intervention description	Costing methodology	Costing type Full vs partial	Reference price	Data collection Retrospective vs prospective	Perspective	Unit costs in 2023 I$
26	Bijlmakers, 2019[10]	Zambia	LMIC	Obstetrics and Gynaecology	Caesarean section	Caesarean section (Livingstone CH)	Mixed	Full	Hospital procurement costs	Retrospective	Hospital	4,785.41
27		Zambia	LMIC	General Surgery	Caesarean section	Caesarean section (Maamba GH)	Mixed	Full	Hospital procurement costs	Retrospective	Hospital	3,071.91
28		Zambia	LMIC	General Surgery	Hernia repair	Hernia repair (Maamba GH)	Mixed	Full	Hospital procurement costs	Retrospective	Hospital	3,354.36
29		Zambia	LMIC	General Surgery	Hernia repair	Hernia repair (Livingstone CH)	Mixed	Full	Hospital procurement costs	Retrospective	Hospital	4,906.46
30	Bocoli, 2013[11]	Brazil	UMIC	Plastic Surgery	Skin carcinoma treatment	Surgical treatment of skin carcinoma	Bottom up	Partial	Hospital billing system	Retrospective	Hospital	411.93
31	Carlos, 2009[12]	Mexico	UMIC	Orthopaedic Surgery	Hip fracture repair	Osteoporosis-related hip fracture repair (INR)	Mixed	Full	Hospital billing system	Prospective	Hospital	4,009.14
32		Mexico	UMIC	Orthopaedic Surgery	Hip fracture repair	Osteoporosis-related hip fracture repair (SPSS)	Mixed	Full	Hospital billing system	Prospective	Hospital	7,383.92
33	Chatterjee, 2013[13]	India	LMIC	Orthopaedic Surgery	Amputation	Amputation	Mixed	Full	mixed	Retrospective	Hospital	433.68
34		India	LMIC	General Surgery	Appendectomy	Appendectomy	Mixed	Full	mixed	Retrospective	Hospital	203.55
35		India	LMIC	General Surgery	Appendectomy	Appendectomy	Mixed	Full	mixed	Retrospective	Hospital	304.03
36		India	LMIC	Interventional Cardiology	Cardiac revascularisation	CABG	Mixed	Full	mixed	Retrospective	Hospital	14,893.72
37		India	LMIC	Obstetrics and Gynaecology	Caesarean section	Caesarean section	Mixed	Full	mixed	Retrospective	Hospital	3,454.53
38		India	LMIC	Obstetrics and Gynaecology	Caesarean section	Caesarean section with tubal ligation	Mixed	Full	mixed	Retrospective	Hospital	3,533.39
39		India	LMIC	Neurosurgery	Craniotomy	Craniotomy	Mixed	Full	mixed	Retrospective	Hospital	6,388.44

S/N	Author last name, year	Country	Income group	Specialty	Broad intervention group	Specific intervention description	Costing methodology	Costing type Full vs partial	Reference price	Data collection Retrospective vs prospective	Perspective	Unit costs in 2023 I$
40		India	LMIC	Orthopaedic Surgery	Long bone fracture fixation	External fracture fixation	Mixed	Full	mixed	Retrospective	Hospital	706.76
41		India	LMIC	ENT	Sinus surgery	Functional endoscopic sinus surgery	Mixed	Full	mixed	Retrospective	Hospital	4,489.62
42		India	LMIC	General Surgery	Hernia repair	Hernia repair	Mixed	Full	mixed	Retrospective	Hospital	1,110.17
43		India	LMIC	Obstetrics and Gynaecology	Hysterectomy	Hysterectomy	Mixed	Full	mixed	Retrospective	Hospital	4,844.76
44		India	LMIC	Obstetrics and Gynaecology	Hysterectomy	Hysterectomy	Mixed	Full	mixed	Retrospective	Hospital	346.74
45		India	LMIC	Obstetrics and Gynaecology	Hysterectomy	Hysterectomy	Mixed	Full	mixed	Retrospective	Hospital	847.59
46		India	LMIC	General Surgery	Intestinal obstruction surgery	Intestinal obstruction surgery	Mixed	Full	mixed	Retrospective	Hospital	538.61
47		India	LMIC	General Surgery	Cholecystectomy	Laparoscopic cholecystectomy	Mixed	Full	mixed	Retrospective	Hospital	2,331.66
48		India	LMIC	Obstetrics and Gynaecology	Caesarean section	Low section caesarean section	Mixed	Full	mixed	Retrospective	Hospital	207.59
49		India	LMIC	Obstetrics and Gynaecology	Caesarean section	Low section caesarean section	Mixed	Full	mixed	Retrospective	Hospital	671.37
50		India	LMIC	General Surgery	Cholecystectomy	Open cholecystectomy	Mixed	Full	mixed	Retrospective	Hospital	3,711.39
51	Cornelissen, 2018[14]	Malawi	LIC	Obstetrics and Gynaecology	Caesarean section	Caesarean section (Mangochi DH)	Top down	Full	Hospital procurement costs	Retrospective	Hospital	226.67
52		Malawi	LIC	Obstetrics and Gynaecology	Caesarean section	Caesarean section (Mulanje DH)	Top down	Full	Hospital procurement costs	Retrospective	Hospital	346.91
53		Malawi	LIC	Obstetrics and Gynaecology	Caesarean section	Caesarean section (Nsanje DH)	Top down	Full	Hospital procurement costs	Retrospective	Hospital	881.80

S/N	Author last name, year	Country	Income group	Specialty	Broad intervention group	Specific intervention description	Costing methodology	Costing type Full vs partial	Reference price	Data collection Retrospective vs prospective	Perspective	Unit costs in 2023 I$
54		Malawi	LIC	General Surgery	Hernia repair	Hernia repair (Mangochi DH)	Top down	Full	Hospital procurement costs	Retrospective	Hospital	189.35
55		Malawi	LIC	General Surgery	Hernia repair	Hernia repair (Mulanje DH)	Top down	Full	Hospital procurement costs	Retrospective	Hospital	226.67
56		Malawi	LIC	General Surgery	Hernia repair	Hernia repair (Nsanje DH)	Top down	Full	Hospital procurement costs	Retrospective	Hospital	826.51
57		Malawi	LIC	Orthopaedic Surgery	Injury surgical treatment	Injury surgical treatment (Mangochi DH)	Top down	Full	Hospital procurement costs	Retrospective	Hospital	203.90
58		Malawi	LIC	Orthopaedic Surgery	Injury surgical treatment	Injury surgical treatment (Mulanje DH)	Top down	Full	Hospital procurement costs	Retrospective	Hospital	238.02
59		Malawi	LIC	Orthopaedic Surgery	Injury surgical treatment	Injury surgical treatment (Nsanje DH)	Top down	Full	Hospital procurement costs	Retrospective	Hospital	719.40
60	Da Silva, 2017[15]	Brazil	UMIC	Cardiothoracic Surgery	Cardiac revascularisation	CABG	Top down	Full	Hospital procurement costs	Retrospective	Hospital	13,839.00
61	Entringer, 2019[16]	Brazil	UMIC	Obstetrics and Gynaecology	Caesarean section	Elective caesarean section	Mixed	Full	National reference price	Interviews	Hospital	1,263.11
62	Evans, 2015[17]	Malawi	LIC	Ophthalmology	Cataract surgery	Cataract surgery (Malawi)	Bottom up	Full	Hospital procurement costs	Prospective	Hospital	599.92
63		Zambia	LMIC	Ophthalmology	Cataract surgery	Cataract surgery (Zambia)	Bottom up	Full	Hospital procurement costs	Prospective	Hospital	618,098.16
64	Falase, 2013[18]	Nigeria	LMIC	Cardiothoracic Surgery	Congenital heart surgery	Atrial septal defect repair	Bottom up	Partial	Hospital billing system	Prospective	Hospital	16,677.56

S/N	Author last name, year	Country	Income group	Specialty	Broad intervention group	Specific intervention description	Costing methodology	Costing type Full vs partial	Reference price	Data collection Retrospective vs prospective	Perspective	Unit costs in 2023 I$
65		Nigeria	LMIC	Cardiothoracic Surgery	Cardiac valve repair	Mitral valve replacement with a bioprosthetic valve	Bottom up	Partial	Hospital billing system	Prospective	Hospital	29,982.13
66		Nigeria	LMIC	Cardiothoracic Surgery	Cardiac revascularisation	Off pump CABG	Bottom up	Partial	Hospital billing system	Prospective	Hospital	22,566.91
67	Fang, 2011[19]	China	UMIC	Ophthalmology	Cataract surgery	Phacoemulsification (FHJ hospital)	Bottom up	Partial	Hospital billing system	Retrospective	Hospital	2,484.92
68		China	UMIC	Ophthalmology	Cataract surgery	Phacoemulsification (JCH hospital)	Bottom up	Partial	Hospital billing system	Retrospective	Hospital	1,439.91
69		China	UMIC	Ophthalmology	Cataract surgery	Phacoemulsification (UH hospital)	Bottom up	Partial	Hospital billing system	Retrospective	Hospital	3,473.51
70		China	UMIC	Ophthalmology	Cataract surgery	Phacoemulsification (ZOC hospital)	Bottom up	Partial	Hospital billing system	Retrospective	Hospital	2,925.49
71	Filho, 2005[20]	Brazil	UMIC	Ophthalmology	Cataract surgery	Phacoemulsification with IOL implantation	Bottom up	Full	Hospital billing system	Prospective	Hospital	1,093.58
72	George, 2017[21]	South Africa	UMIC	Paediatric Surgery	Male circumcision	Voluntary medical male circumcision	Mixed	Full	Local market prices	Retrospective	Hospital	113.60
73	Glaeser, 2020[22]	Cambodia	LMIC	Obstetrics and Gynaecology	Caesarean section	Caesarean section in a government hospital	Mixed	Full	Hospital procurement costs	Prospective	Hospital	2,241.91
74		Cambodia	LMIC	Obstetrics and Gynaecology	Caesarean section	Caesarean section in a non-governmental hospital	Mixed	Full	Hospital procurement costs	Prospective	Hospital	1,542.33

S/N	Author last name, year	Country	Income group	Specialty	Broad intervention group	Specific intervention description	Costing methodology	Costing type Full vs partial	Reference price	Data collection Retrospective vs prospective	Perspective	Unit costs in 2023 I$
75	Gogate, 2010[23]	India	LMIC	Ophthalmology	Cataract surgery	Paediatric cataract surgery	Top down	Full	Hospital procurement costs	Retrospective	Hospital	1,062.81
76	Goldraich, 2018[24]	Brazil	UMIC	Transplant Surgery	Heart transplant	Heart transplant	Bottom up	Full	Hospital billing system	Prospective	Hospital	167,393.64
77	Griffiths, 2015[25]	Zambia	LMIC	Ophthalmology	Cataract surgery	Cataract surgery	Mixed	Full	Hospital reference prices	Retrospective	Hospital	216,648.80
78		Zambia	LMIC	Ophthalmology	Refractive surgery	Refractive surgery	Mixed	Full	Hospital reference prices	Retrospective	Hospital	164,948.52
79	Gungor, 2021[26]	Turkiye	UMIC	Orthopaedic Surgery	Knee arthroplasty	Primary total knee arthroplasty	Bottom up	Partial	Hospital billing system	Retrospective	Hospital	6,536.30
80		Turkiye	UMIC	Orthopaedic Surgery	Knee arthroplasty	Revised total knee arthroplasty	Bottom up	Partial	Hospital billing system	Retrospective	Hospital	21,084.43
81	Habtamu, 2014[27]	Ethiopia	LIC	Ophthalmology	Cataract surgery	Cataract surgery	Mixed	Full	Hospital procurement costs	Interviews	Hospital	688.53
82	Haddad, 2007[28]	Brazil	UMIC	Cardiothoracic Surgery	Cardiac revascularisation	CABG	Bottom up	Full	Hospital reference prices	Prospective	Hospital	10,046.79
83	Haldar, 2021[29]	India	LMIC	Obstetrics and Gynaecology	Hysterectomy	Abdominal hysterectomy	Bottom up	Full	Hospital billing system	Retrospective	Hospital	1,250.92
84		India	LMIC	Obstetrics and Gynaecology	Caesarean section	Caesarean section	Bottom up	Full	Hospital billing system	Retrospective	Hospital	1,068.09
85		India	LMIC	Obstetrics and Gynaecology	Hysterectomy	Vaginal hysterectomy	Bottom up	Full	Hospital billing system	Retrospective	Hospital	1,167.24

S/N	Author last name, year	Country	Income group	Specialty	Broad intervention group	Specific intervention description	Costing methodology	Costing type Full vs partial	Reference price	Data collection Retrospective vs prospective	Perspective	Unit costs in 2023 I$
86	He, 2023[30]	China	UMIC	Cardiothoracic Surgery	Congenital heart surgery	Congenital heart surgery (median cost)	Bottom up	Partial	Hospital billing system	Retrospective	Hospital	18,879.31
87	Helal, 2019[31]	Egypt	LMIC	Neurosurgery	Craniotomy	Craniotomy for tumour resection	Bottom up	Partial	Hospital billing system	Retrospective	Hospital	11,694.33
88	Hepguler, 2012[32]	Turkiye	UMIC	Orthopaedic Surgery	Hip fracture repair	Hip joint replacement	Bottom up	Partial	Hospital billing system	Retrospective	Hospital	7,460.39
89		Turkiye	UMIC	Orthopaedic Surgery	Hip fracture repair	Internal/external fixation	Bottom up	Partial	Hospital billing system	Retrospective	Hospital	5,138.89
90		Turkiye	UMIC	Orthopaedic Surgery	Hip fracture repair	Other hip and femur procedures	Bottom up	Partial	Hospital billing system	Retrospective	Hospital	5,105.36
91	Hofmeyr, 2016[33]	South Africa	UMIC	General Surgery	Bile duct injury repair	Laparoscopic bile duct injury repair	Bottom up	Partial	Hospital billing system	Retrospective	Hospital	49,965.34
92	Huang, 2006[34]	China	UMIC	Interventional Cardiology	Catheter ablation	Radiofrequency catheter ablation for premature ventricular contractions	Bottom up	Partial	Hospital billing system	Retrospective	Hospital	7,930.27
93	Ifeanyichi, 2021[35]	Tanzania	LMIC	ENT	Adenectomy	Adenectomy (Mt Meru RH)	Mixed	Full	mixed	Retrospective	Hospital	1,330.81
94		Tanzania	LMIC	ENT	Adenoidectomy	Adenotonsillectomy (Mt Meru RH)	Mixed	Full	mixed	Retrospective	Hospital	1,510.75
95		Tanzania	LMIC	General Surgery	Appendectomy	Appendectomy (Meru DH)	Mixed	Full	mixed	Retrospective	Hospital	652.28
96		Tanzania	LMIC	General Surgery	Appendectomy	Appendectomy (Mt Meru RH)	Mixed	Full	mixed	Retrospective	Hospital	716.01
97		Tanzania	LMIC	Obstetrics and Gynaecology	Caesarean section	Caesarean Section (Meru DH)	Mixed	Full	mixed	Retrospective	Hospital	775.99

S/N	Author last name, year	Country	Income group	Specialty	Broad intervention group	Specific intervention description	Costing methodology	Costing type Full vs partial	Reference price	Data collection Retrospective vs prospective	Perspective	Unit costs in 2023 I$
98		Tanzania	LMIC	Obstetrics and Gynaecology	Caesarean section	Caesarean Section (Mt Meru RH)	Mixed	Full	mixed	Retrospective	Hospital	446.10
99		Tanzania	LMIC	Obstetrics and Gynaecology	Caesarean section	Caesarean Section (Oltrumet DH)	Mixed	Full	mixed	Retrospective	Hospital	738.51
100		Tanzania	LMIC	General Surgery	Hernia repair	Hernia repair (Meru DH)	Mixed	Full	mixed	Retrospective	Hospital	1,248.34
101		Tanzania	LMIC	General Surgery	Hernia repair	Hernia repair (Mt Meru RH)	Mixed	Full	mixed	Retrospective	Hospital	1,750.67
102		Tanzania	LMIC	General Surgery	Hernia repair	Hernia repair (Oltrumet DH)	Mixed	Full	mixed	Retrospective	Hospital	671.03
103		Tanzania	LMIC	Urology	Hydrocelectomy	hydrocoelectomy (Meru DH)	Mixed	Full	mixed	Retrospective	Hospital	607.30
104		Tanzania	LMIC	Obstetrics and Gynaecology	Hysterectomy	Hysterectomy (Mt Meru RH)	Mixed	Full	mixed	Retrospective	Hospital	895.95
105		Tanzania	LMIC	General Surgery	Laparotomy	Laparotomy (Mt Meru RH)	Mixed	Full	mixed	Retrospective	Hospital	1,893.12
106		Tanzania	LMIC	Obstetrics and Gynaecology	Myomectomy	Myomectomy (Meru DH)	Mixed	Full	mixed	Retrospective	Hospital	1,559.48
107		Tanzania	LMIC	Obstetrics and Gynaecology	Myomectomy	Myomectomy (Mt Meru RH)	Mixed	Full	mixed	Retrospective	Hospital	1,334.56
108		Tanzania	LMIC	Obstetrics and Gynaecology	Myomectomy	Myomectomy (Oltrumet DH)	Mixed	Full	mixed	Retrospective	Hospital	1,889.37
109		Tanzania	LMIC	Orthopaedic Surgery	Long bone fracture fixation	Open reduction internal fixation (Mt Meru RH)	Mixed	Full	mixed	Retrospective	Hospital	2,316.73
110		Tanzania	LMIC	Obstetrics and Gynaecology	Salpingectomy	Salpingectomy (Meru DH)	Mixed	Full	mixed	Retrospective	Hospital	689.77
111		Tanzania	LMIC	Obstetrics and Gynaecology	Salpingectomy	Salpingectomy (Mt Meru RH)	Mixed	Full	mixed	Retrospective	Hospital	341.14
112		Tanzania	LMIC	Orthopaedic Surgery	Surgical debridement	Surgical debridement (Mt Meru RH)	Mixed	Full	mixed	Retrospective	Hospital	4,412.29

S/N	Author last name, year	Country	Income group	Specialty	Broad intervention group	Specific intervention description	Costing methodology	Costing type Full vs partial	Reference price	Data collection Retrospective vs prospective	Perspective	Unit costs in 2023 I$
113		Tanzania	LMIC	ENT	Tonsillectomy	Tonsillectomy (Mt Meru RH)	Mixed	Full	mixed	Retrospective	Hospital	1,330.81
114	Jiang, 2020[36]	China	UMIC	Orthopaedic Surgery	Orthopaedic infection treatment	Surgical treatment of traumatic extremity osteomielitis	Bottom up	Partial	Hospital billing system	Retrospective	Hospital	21,494.53
115	Jones, 2020[37]	Mexico	UMIC	Interventional Cardiology	Cardiac revascularisation	Percutaneous coronary intervention	Bottom up	Full	Hospital reference prices	Retrospective	Hospital	19,161.14
116	Kaur, 2022[38]	India	LMIC	Interventional Cardiology	Cardiac defibrillator implantation	Same day PCI	Bottom up	Full	Hospital billing system	Prospective	Hospital	2,910.03
117	Kihuba, 2022[39]	Kenya	LMIC	Orthopaedic Surgery	Ankle & foot procedures	Ankle & foot procedures	Bottom up	Partial	Hospital billing system	Retrospective	Hospital	3,673.88
118		Kenya	LMIC	Orthopaedic Surgery	Elbow, shoulder and knee repairs	Elbow, shoulder and knee repairs	Bottom up	Partial	Hospital billing system	Retrospective	Hospital	3,947.40
119		Kenya	LMIC	Orthopaedic Surgery	Limb deformities procedures	Limb deformities procedures	Bottom up	Partial	Hospital billing system	Retrospective	Hospital	3,267.96
120		Kenya	LMIC	Orthopaedic Surgery	Orthopaedic tumour surgery	Orthopaedic tumour surgery	Bottom up	Partial	Hospital billing system	Retrospective	Hospital	2,126.45
121		Kenya	LMIC	Orthopaedic Surgery	Orthopaedic infection treatment	Orthopaedic infection treatment	Bottom up	Partial	Hospital billing system	Retrospective	Hospital	4,024.10
122		Kenya	LMIC	Orthopaedic Surgery	Injury surgical treatment	Orthopaedic injury surgical treatment	Bottom up	Partial	Hospital billing system	Retrospective	Hospital	4,283.58
123		Kenya	LMIC	Orthopaedic Surgery	Removal of orthopaedic hardware	Removal of orthopaedic hardware	Bottom up	Partial	Hospital billing system	Retrospective	Hospital	3,376.48

S/N	Author last name, year	Country	Income group	Specialty	Broad intervention group	Specific intervention description	Costing methodology	Costing type Full vs partial	Reference price	Data collection Retrospective vs prospective	Perspective	Unit costs in 2023 I$
124		Kenya	LMIC	Orthopaedic Surgery	Hip fracture repair	Total hip arthroplasty	Bottom up	Partial	Hospital billing system	Retrospective	Hospital	9,440.79
125		Kenya	LMIC	Orthopaedic Surgery	Knee arthroplasty	Total knee arthroplasty	Bottom up	Partial	Hospital billing system	Retrospective	Hospital	11,093.14
126	Lee, 2018[40]	Malaysia	UMIC	Interventional Cardiology	Cardiac revascularisation	Elective PCI	Top down	Full	Hospital procurement costs	Retrospective	Hospital	10,707.53
127	Nourbala, 2007[41]	Iran	UMIC	Transplant Surgery	Kidney transplant	Kidney transplantation from living donor	Bottom up	Partial	Hospital billing system	Retrospective	Hospital	6,573.55
128	Odhiambo, 2019[42]	Rwanda	LIC	Obstetrics and Gynaecology	Caesarean section	Emergency caesarean section	Bottom up	Full	Hospital procurement costs	Retrospective	Hospital	1,154.69
129	Ouakhzan, 2020[43]	Morrocco	LMIC	Obstetrics and Gynaecology	Caesarean section	Caesarean section	Bottom up	Full	Hospital reference prices	Mixed	Hospital	880.96
130	Pejcic, 2018[44]	Serbia	UMIC	Orthopaedic Surgery	Knee arthroplasty	Bilateral total knee replacement	Bottom up	Partial	Hospital billing system	Retrospective	Hospital	14,412.01
131		Serbia	UMIC	Orthopaedic Surgery	Knee arthroplasty	Unilateral total knee replacement	Bottom up	Partial	Hospital billing system	Retrospective	Hospital	8,140.68
132	Portela, 2010[45]	Brazil	UMIC	Transplant Surgery	Liver transplant	Liver transplant	Bottom up	Full	Hospital reference prices	Retrospective	Hospital	38,573.83
133	Prinja, 2019[46]	India	LMIC	Cardiothoracic Surgery	Cardiac valve repair	Balloon valvotomy	Bottom up	Full	National reference price	Retrospective	Hospital	563.02
134		India	LMIC	Cardiothoracic Surgery	Cardiac valve repair	Double valve replacement	Bottom up	Full	National reference price	Retrospective	Hospital	5,132.02

S/N	Author last name, year	Country	Income group	Specialty	Broad intervention group	Specific intervention description	Costing methodology	Costing type Full vs partial	Reference price	Data collection Retrospective vs prospective	Perspective	Unit costs in 2023 I$
135		India	LMIC	Cardiothoracic Surgery	Cardiac valve repair	Single valve replacement	Bottom up	Full	National reference price	Retrospective	Hospital	4,902.68
136		India	LMIC	Cardiothoracic Surgery	Cardiac valve repair	Triple valve replacement	Bottom up	Full	National reference price	Retrospective	Hospital	5,992.28
137	Qin, 2021[47]	China	UMIC	Cardiothoracic Surgery	Cardiac valve repair	Aortic valve replacement	Top down	Full	Hospital billing system	Retrospective	Hospital	39,672.96
138	Rahmawati, 2021[48]	Indonesia	UMIC	Obstetrics and Gynaecology	Caesarean section	Caesarean section	Mixed	Full	mixed	Retrospective	Hospital	566.98
139	Rashidian, 2018[49]	Iran, Islamic Rep.	UMIC	Obstetrics and Gynaecology	Caesarean section	Caesarean section (Hospital A)	Bottom up	Partial	Hospital billing system	Retrospective	Hospital	813.36
140		Iran, Islamic Rep.	UMIC	Obstetrics and Gynaecology	Caesarean section	Caesarean section (Hospital A)	Bottom up	Partial	Hospital billing system	Retrospective	Hospital	599.09
141		Iran, Islamic Rep.	UMIC	Obstetrics and Gynaecology	Caesarean section	Caesarean section (Hospital B)	Bottom up	Partial	Hospital billing system	Retrospective	Hospital	561.65
142		Iran, Islamic Rep.	UMIC	Obstetrics and Gynaecology	Caesarean section	Caesarean section (Hospital B)	Bottom up	Partial	Hospital billing system	Retrospective	Hospital	480.77
143	Rizal, 2004[50]	Malaysia	UMIC	Ophthalmology	Cataract surgery	Extracapsular cataract extraction	Mixed	Full	mixed	Prospective	Hospital	2,127.64
144		Malaysia	UMIC	Ophthalmology	Cataract surgery	Phacoemulsification	Mixed	Full	mixed	Prospective	Hospital	2,528.32
145	Ruhumuriza, 2018[51]	Rwanda	LIC	General Surgery	Laparotomy	Laparotomy at district hospitals	Bottom up	Full	Hospital procurement costs	Prospective	Hospital	3,662.36
146	Saldarriaga, 2021[52]	Peru	UMIC	Obstetrics and Gynaecology	Genital warts treatment	Genital warts electrosurgery	Bottom up	Full	mixed	Prospective	Hospital	42.00

S/N	Author last name, year	Country	Income group	Specialty	Broad intervention group	Specific intervention description	Costing methodology	Costing type Full vs partial	Reference price	Data collection Retrospective vs prospective	Perspective	Unit costs in 2023 I$
147		Peru	UMIC	Obstetrics and Gynaecology	Genital warts treatment	Genital warts excision	Bottom up	Full	mixed	Prospective	Hospital	59.22
148		Peru	UMIC	Obstetrics and Gynaecology	Genital warts treatment	Genital warts cryotherapy	Bottom up	Full	mixed	Prospective	Hospital	37.09
149	Salgado Júnior, 2011[53]	Brazil	UMIC	General Surgery	Bariatric surgery	Bariatric surgery	Bottom up	Partial	Hospital billing system	Retrospective	Hospital	6,837.09
150		Brazil	UMIC	General Surgery	Bariatric surgery	Bariatric surgery	Bottom up	Partial	Hospital billing system	Retrospective	Hospital	7,516.76
151	Salzedas-Netto, 2015[54]	Brazil	UMIC	Transplant Surgery	Simultaneous pancreas-kidney transplantation	Simultaneous pancreas-kidney transplantation (supplementary system)	Bottom up	Partial	Hospital billing system	Prospective	Hospital	41,632.74
152		Brazil	UMIC	Transplant Surgery	Simultaneous pancreas-kidney transplantation	Simultaneous pancreas-kidney transplantation (sus)	Bottom up	Partial	Hospital billing system	Prospective	Hospital	32,979.54
153	Sangwan, 2018[55]	India	LMIC	Orthopaedic Surgery	Injury surgical treatment	Injury surgical treatment (major surgery DH)	Mixed	Full	mixed	Prospective	Hospital	421.44
154		India	LMIC	Orthopaedic Surgery	Injury surgical treatment	Injury surgical treatment (minor surgery DH)	Mixed	Full	mixed	Prospective	Hospital	12.05
155		India	LMIC	Orthopaedic Surgery	Injury surgical treatment	Injury surgical treatment (TH)	Mixed	Full	mixed	Prospective	Hospital	1,392.86
156	Sarowar, 2010[56]	Bangladesh	LMIC	Obstetrics and Gynaecology	Caesarean section	Complicated caesarean section	Mixed	Full	Hospital procurement costs	Retrospective	Hospital	932.08
157		Bangladesh	LMIC	Obstetrics and Gynaecology	Caesarean section	Non-complicated caesarean	Mixed	Full	Hospital procurement costs	Retrospective	Hospital	716.09

S/N	Author last name, year	Country	Income group	Specialty	Broad intervention group	Specific intervention description	Costing methodology	Costing type Full vs partial	Reference price	Data collection Retrospective vs prospective	Perspective	Unit costs in 2023 I$
158	Sekeitto, 2021[57]	South Africa	UMIC	Orthopaedic Surgery	Hip fracture repair	Total hip arthroplasty	Bottom up	Partial	Hospital billing system	Retrospective	Hospital	11,158.02
159	Sheriff, 2018[58]	Sudan	LIC	Plastic Surgery	Oral and maxillofacial reconstruction	Cleft surgery	Bottom up	Partial	Local market prices	Retrospective	Hospital	1,046.36
160		Palestine	LMIC	Plastic Surgery	Oral and maxillofacial reconstruction	Cleft surgery	Bottom up	Partial	Local market prices	Retrospective	Hospital	5,921.19
161	Shetty, 2021[59]	India	LMIC	Obstetrics and Gynaecology	Surgical treatment of atonic PPH	Devascularisation	Bottom up	Full	mixed	Retrospective	Hospital	257.81
162		India	LMIC	Obstetrics and Gynaecology	Hysterectomy	Hysterectomy for atonic PPH	Bottom up	Full	mixed	Retrospective	Hospital	412.50
163	Singh, 2022[60]	India	LMIC	ENT	Adenoidectomy	Adenoidectomy	Mixed	Full	mixed	Retrospective	Hospital	313.19
164		India	LMIC	Orthopaedic Surgery	Arthrodesis	Ankle/triple arthrodesis without implant	Mixed	Full	mixed	Retrospective	Hospital	1,083.81
165		India	LMIC	Ophthalmology	Capsulotomy	Capsulotomy	Mixed	Full	mixed	Retrospective	Hospital	94.78
166		India	LMIC	Obstetrics and Gynaecology	Caesarean section	Caesarean section	Mixed	Full	mixed	Retrospective	Hospital	717.04
167		India	LMIC	Orthopaedic Surgery	Fasciectomy	Dupuytren's contracture release and rehabilitation	Mixed	Full	mixed	Retrospective	Hospital	527.48
168		India	LMIC	Obstetrics and Gynaecology	Electrosurgery	Electrosurgery and cryosurgery	Mixed	Full	mixed	Retrospective	Hospital	247.26
169		India	LMIC	ENT	Sinus surgery	Endoscopic sinus surgery	Mixed	Full	mixed	Retrospective	Hospital	1,203.31
170		India	LMIC	Orthopaedic Surgery	Hip fracture repair	Femur's neck fracture closed reduction and	Mixed	Full	mixed	Retrospective	Hospital	964.30

S/N	Author last name, year	Country	Income group	Specialty	Broad intervention group	Specific intervention description	Costing methodology	Costing type Full vs partial	Reference price	Data collection Retrospective vs prospective	Perspective	Unit costs in 2023 I$
						percutaneous screw fixation						
171		India	LMIC	ENT	Sinus surgery	Functional endoscopic sinus surgery	Mixed	Full	mixed	Retrospective	Hospital	684.08
172		India	LMIC	General Surgery	Haemorrhoidectomy	Haemorrhoidectomy without stapler	Mixed	Full	mixed	Retrospective	Hospital	935.45
173		India	LMIC	General Surgery	Cutaneous swellings excision	Lipoma/cyst/other cutaneous swellings excision	Mixed	Full	mixed	Retrospective	Hospital	313.19
174		India	LMIC	Obstetrics and Gynaecology	Appendectomy	Open appendectomy	Mixed	Full	mixed	Retrospective	Hospital	1,228.04
175		India	LMIC	Ophthalmology	Cataract surgery	Phacoemulsification with foldable hydrophobic acrylic IOL	Mixed	Full	mixed	Retrospective	Hospital	280.22
176		India	LMIC	Ophthalmology	Pterygium surgery	Pterygium surgery and conjunctival autograft	Mixed	Full	mixed	Retrospective	Hospital	576.93
177		India	LMIC	General Surgery	Mastectomy	Radical or modified radical mastectomy	Mixed	Full	mixed	Retrospective	Hospital	1,557.71
178		India	LMIC	ENT	Septoplasty	Septoplasty	Mixed	Full	mixed	Retrospective	Hospital	745.89
179		India	LMIC	Ophthalmology	Cataract surgery	SICS with non-foldable IOL	Mixed	Full	mixed	Retrospective	Hospital	247.26
180		India	LMIC	Obstetrics and Gynaecology	Hysterectomy	Total laparoscopic hysterectomy	Mixed	Full	mixed	Retrospective	Hospital	1,244.52
181		India	LMIC	General Surgery	Thyroidectomy	Total thyroidectomy	Mixed	Full	mixed	Retrospective	Hospital	1,244.52
182		India	LMIC	Ophthalmology	Glaucoma surgery	Trabeculectomy with or without mitomycin c	Mixed	Full	mixed	Retrospective	Hospital	704.68
183		India	LMIC	ENT	Tympanoplasty	Tympanoplasty	Mixed	Full	mixed	Retrospective	Hospital	803.58

S/N	Author last name, year	Country	Income group	Specialty	Broad intervention group	Specific intervention description	Costing methodology	Costing type Full vs partial	Reference price	Data collection Retrospective vs prospective	Perspective	Unit costs in 2023 I$
184		India	LMIC	Obstetrics and Gynaecology	Hysterectomy	Vaginal hysterectomy and colpoperineorrhaphy	Mixed	Full	mixed	Retrospective	Hospital	1,244.52
185	Singh, 2021[61]	India	LMIC	Obstetrics and Gynaecology	Hysterectomy	Radical hysterectomy	Mixed	Full	mixed	Retrospective	Hospital	1,260.63
186	Soriano, 2022[62]	Brazil	UMIC	Neurosurgery	Hydrocephalus surgery	Endoscopic third ventriculostomy	Bottom up	Partial	Hospital billing system	Retrospective	Hospital	1,102.44
187		Brazil	UMIC	Neurosurgery	Hydrocephalus surgery	Ventriculoperitoneal shunt	Bottom up	Partial	Hospital billing system	Retrospective	Hospital	2,291.30
188	Souza, 2019[63]	Brazil	UMIC	Transplant Surgery	Liver transplant	Adult liver transplant	Mixed	Full	Hospital billing system	Retrospective	Hospital	37,196.28
189	Taazan, 2021[64]	Mongolia	UMIC	Obstetrics and Gynaecology	Caesarean section	Caesarean section	Top down	Full	Hospital procurement costs	Retrospective	Hospital	2,933.09
190	Umo, 2021[65]	Papua New Guinea	LMIC	General Surgery	Appendectomy	Appendectomy (complicated appendicitis)	Bottom up	Full	National reference price	Prospective	Hospital	25,099.15
191		Papua New Guinea	LMIC	General Surgery	Appendectomy	Appendectomy (uncomplicated appendicitis)	Bottom up	Full	National reference price	Prospective	Hospital	21,621.39
192		Papua New Guinea	LMIC	General Surgery	Appendectomy	Appendectomy (uninflamed appendix)	Bottom up	Full	National reference price	Prospective	Hospital	21,249.97
193	Van Heukelum, 2021[66]	South Africa	UMIC	Orthopaedic Surgery	Injury surgical treatment	Orthopaedic gunshot-related injury surgery	Bottom up	Partial	Hospital billing system	Retrospective	Hospital	8,744.19
194	von Both, 2008[67]	Tanzania	LMIC	Obstetrics and Gynaecology	Caesarean section	Caesarean section	Top down	Full	Mixed	Retrospective	Hospital	1.54

S/N	Author last name, year	Country	Income group	Specialty	Broad intervention group	Specific intervention description	Costing methodology	Costing type Full vs partial	Reference price	Data collection Retrospective vs prospective	Perspective	Unit costs in 2023 I$
195	Wang, 2019[68]	China	UMIC	Orthopaedic Surgery	Hip fracture repair	Hip replacement	Bottom up	Partial	Hospital billing system	Retrospective	Hospital	18,333.76
196		China	UMIC	Orthopaedic Surgery	Hip fracture repair	Internal fixation	Bottom up	Partial	Hospital billing system	Retrospective	Hospital	13,604.86
197		China	UMIC	Orthopaedic Surgery	Hip fracture repair	Other hip fracture procedures	Bottom up	Partial	Hospital billing system	Retrospective	Hospital	352.35
198	Yang, 2019a[69]	China	UMIC	Interventional Cardiology	Cardiac revascularisation	High risk CABG	Bottom up	Partial	Hospital billing system	Retrospective	Hospital	2,495.15
199		China	UMIC	Interventional Cardiology	Cardiac revascularisation	Low risk CABG	Bottom up	Partial	Hospital billing system	Retrospective	Hospital	2,586.38
200	Yang, 2019b[70]	China	UMIC	Plastic Surgery	Oral and maxillofacial reconstruction	Oral and maxillofacial free flap reconstruction	Bottom up	Partial	Hospital billing system	Retrospective	Hospital	5,732.90
201	Yereli, 2009[71]	Turkiye	UMIC	General Surgery	Cholecystectomy	Laparoscopic cholecystectomy (activity-based costing)	Mixed	Full	Hospital procurement costs	Retrospective	Hospital	1,395.57
202		Turkiye	UMIC	General Surgery	Cholecystectomy	Laparoscopic cholecystectomy (traditional costing approach)	Mixed	Full	Hospital procurement costs	Retrospective	Hospital	1,244.78
203		Turkiye	UMIC	General Surgery	Cholecystectomy	Open cholecystectomy (activity-based costing)	Mixed	Full	Hospital procurement costs	Retrospective	Hospital	2,246.88
204		Turkiye	UMIC	General Surgery	Cholecystectomy	Open cholecystectomy	Mixed	Full	Hospital procurement costs	Retrospective	Hospital	2,451.95

S/N	Author last name, year	Country	Income group	Specialty	Broad intervention group	Specific intervention description	Costing methodology	Costing type Full vs partial	Reference price	Data collection Retrospective vs prospective	Perspective	Unit costs in 2023 I$
						(traditional costing approach)						
205	Younis, 2015[72]	Palestine	LMIC	Transplant Surgery	Kidney transplant	Kidney transplant	Bottom up	Full	Hospital reference prices	Retrospective	Hospital	19,174.31
206	Zhao, 2012[73]	China	UMIC	Transplant Surgery	Kidney transplant	Living-related donor kidney transplantation	Bottom up	Partial	Hospital billing system	Retrospective	Hospital	20,988.00
207	Zhu, 2012[74]	China	UMIC	General Surgery	Hepatectomy	Hepatectomy	Bottom up	Partial	Hospital billing system	Retrospective	Hospital	5,759.00
208		China	UMIC	General Surgery	Hepatectomy	Major hepatectomy for Hepatocellular Carcinoma	Bottom up	Partial	Hospital billing system	Retrospective	Hospital	5,052.80
209		China	UMIC	General Surgery	Hepatectomy	Segmentectomy for Hepatocellular Carcinoma	Bottom up	Partial	Hospital billing system	Retrospective	Hospital	4,501.00
210		China	UMIC	General Surgery	Hepatectomy	Wedge resection for Hepatocellular Carcinoma	Bottom up	Partial	Hospital billing system	Retrospective	Hospital	4,469.00

Abbreviations: CABG: Coronary artery bypass graft; CH: Central hospital; DH: District hospital; GH: General hospital; RH: Regional hospital; ENT: Ear, nose and throat; IOL: Intra-ocular lens; LIC: Low-income country; LMIC: Lower middle-income country; PCI: Percutaneous coronary intervention; PPH: Post-partum haemorrhage; SICS: Small Incision Cataract Surgery; TH: Teaching hospital; UMIC: Upper middle-income country.

References

1. Abdelgadir J, Tran T, Muhindo A, et al. Estimating the Cost of Neurosurgical Procedures in a Low-Income Setting: An Observational Economic Analysis. 2017; **101**: 651-7.
2. Abeygunasekera AM, Duminda MT, Thushara C, Ruwan J. The operational cost of a urology unit. *Ceylon Medical Journal* 2008; **53**(2): 45-8.
3. Ahmadi ZH, Shafaghi S, Mandegar MH, et al. The Cost of Heart Transplant in Iran: A Multicenter Analysis. 2021; **12**: 23-9.
4. Akarsu M, Matur M, Karademir S, Unek T, Astarcioglu I. Cost analysis of liver transplantation in Turkey. 2012; **43**: 3783-8.
5. Aljarrah Q, Bakkar S, Aleshawi A, et al. Analysis of the Peri-Operative Cost of Non-Traumatic Major Lower Extremity Amputation in Jordan. 2020; **12**: 13-21.
6. Allard D, Burch VC. The cost of treating serious abdominal firearm-related injuries in South Africa. 2005; **95**: 591-4.
7. Balakrishnan K, Nageswaran C. Cost analysis of 163 consecutive heart transplant operationsin an Indian setting. 2020; **36**: 200-9.
8. Carvalho Barreto MF, Gomes Dellaroza MS, Parron Fernandes KB, de Souza Cavalcante Pissinati P, Quina Galdino MJ, Lourenco Haddad M. Cost and Factors Associated With the Hospitalization of Patients Undergoing Heart Transplantation. 2020b; **51**: 3412-7.
9. Carvalho Barreto MF, Dellaroza MSG, Fernandes KBP, Pissinati PSC, Galdino MJQ, Haddad M. Hospitalization costs and their determining factors among patients undergoing kidney transplantation: a cross-sectional descriptive study. 2020a; **137**: 498-504.
10. Bijlmakers L, Cornelissen D, Cheelo M, et al. The cost of providing and scaling up surgery: a comparison of a district hospital and a referral hospital in Zambia. 2019; **33**: 1055-64.
11. Bocoli KH, Veiga DF, Cabral IV, et al. Surgical treatment of skin carcinomas in the Brazilian Unified Health System: costs analysis. 2013; **40**: 449-52.
12. Carlos F, Clark P, Maciel H, Tamayo JA. Direct costs of osteoporosis and hip fracture: an analysis for the Mexican Social Insurance Health Care System. 2009; **51**: S108-13.
13. Chatterjee S, Laxminarayan R. Costs of surgical procedures in Indian hospitals. 2013; **3**.
14. Cornelissen D, Mwapasa G, Gajewski J, et al. The Cost of Providing District-Level Surgery in Malawi. 2018; **42**: 46-53.
15. Da Silva GS, Colosimo FC, Sousa AG, Piotto RF, Castilho V. Coronary Artery Bypass Graft Surgery Cost Coverage by the Brazilian Unified Health System (SUS). 2017; **32**: 253-9.
16. Entringer AP, Pinto MFT, Gomes MASM. Costs analysis of hospital care for vaginal delivery and elective caesarean section for usual risk pregnant women in the Brazilian Unified National Health System. *Ciencia & saude coletiva* 2019; **24**(4): 1527-36.
17. Evans CT, Lenhart PD, Lin D, et al. A cost analysis of pediatric cataract surgery at two child eye health tertiary facilities in Africa. 2015; **18**: 559-62.
18. Falase B, Sanusi M, Majekodunmi A, Ajose I, Idowu A, Oke D. The cost of open heart surgery in Nigeria. 2013; **14**: 61.
19. Fang J, Wang X, Lin Z, Yan J, Yang Y, Li J. Variation of cataract surgery costs in four different graded providers of China. 2011; **10**: 543.
20. Saad Filho R, Loureiro Saad FG, Lemes De Freitas L. Cost of phacoemulsification in the national campaing of elective cataract surgery in Itapolis, SP, Brazil. *Arquivos Brasileiros de Oftalmologia* 2005; **68**(1): 55-9.
21. George G, Strauss M, Asfaw E. The cost of demand creation activities and voluntary medical male circumcision targeting school-going adolescents in KwaZulu-Natal, South Africa. 2017; **12**: e0179854.
22. Glaeser E, Jacobs B, Appelt B, et al. Costing of Cesarean Sections in a Government and a Non-Governmental Hospital in Cambodia-A Prerequisite for Efficient and Fair Comprehensive Obstetric Care. 2020; **17**.
23. Gogate P, Dole K, Ranade S, Deshpande M. Cost of pediatric cataract surgery in Maharashtra, India. 2010; **3**: 182-6.

24. Goldraich LA, Neyeloff JL, da Silva Etges APB, et al. Heart Transplantation Cost Composition in Brazil: A Patient-Level Microcosting Analysis and Comparison With International Data. 2018; **24**: 860-3.
25. Griffiths UK, Bozzani F, Muleya L, Mumba M. Costs of eye care services: prospective study from a faith-based hospital in Zambia. 2015; **22**: 43-51.
26. Gungor E, Basarir K, Binnet MS. A retrospective comparison of costs for the primary and revision total knee arthroplasty in Turkey. 2021; **54**: 541-5.
27. Habtamu E, Eshete Z, Burton MJ. Cataract surgery in Southern Ethiopia: distribution, rates and determinants of service provision. 2014; **13**: 480.
28. Haddad N, Bittar E, de Marchi AF, et al. Hospital costs of coronary artery bypass grafting on elective coronary patients. 2007; **88**: 418-23.
29. Haldar J, Kamath R, Stallone D'lima K, Nazareth J. An Assessment of the Economic Feasibility of Selected Surgeries in the Obstetrics and Gynaecology Department under Community-Based Health Insurance (CBHI) in a Tertiary Care Hospital in South India. 2021; **2021**: 1158533.
30. He Q, Dou Z, Su Z, et al. Inpatient costs of congenital heart surgery in China: results from the National Centre for Cardiovascular Diseases. 2023; **31**: 100623.
31. Helal AE, Abouzahra H, Fayed AA, Rayan T, Abbassy M. Socioeconomic restraints and brain tumor surgery in low-income countries. 2019; **45**: E11.
32. Hepguler S, Cetin A, Deger C, Erkent U. Osteoporotic hip fracture costs in the elderly Turkish population. 2012; **45**: 316-25.
33. Hofmeyr S, Krige JE, Bornman PC, Beningfield SJ. A cost analysis of operative repair of major laparoscopic bile duct injuries. 2016; **105**: 454-7.
34. Huang CX, Liang JJ, Yang B, et al. Quality of life and cost for patients with premature ventricular contractions by radiofrequency catheter ablation. 2006; **29**: 343-50.
35. Ifeanyichi M, Broekhuizen H, Juma A, et al. Economic Costs of Providing District- and Regional-Level Surgeries in Tanzania. 2021.
36. Jiang N, Wu HT, Lin QR, Hu YJ, Yu B. Health Care Costs of Post-traumatic Osteomyelitis in China: Current Situation and Influencing Factors. 2020; **247**: 356-63.
37. Jones K, Ramirez J, Papadimitropoulos M, et al. Resource Utilization and Costs Associated With Percutaneous Coronary Intervention When Treating Patients With Acute Coronary Syndrome in the Mexican National Institute of Cardiology "Ignacio Chavez". 2020; **20**: 136-41.
38. Kaur N, Vijayvergiya R, Prinja S, Dixit J, Sahoo SK, Kasinadhuni G. Economic Impact And SafetY of Same-Day Discharge Following Percutaneous Coronary Intervention: A Tertiary-Care Centre Experience From Northern India (EASY-SDD). 2022; **40**: 71-7.
39. Kihuba E. Epidemiology and medical costs of orthopedic conditions in a tertiary hospital in Kenya; A five-year analysis of admission data. *medRxiv* 2022.
40. Lee KY, Ong TK, Low EV, et al. Cost of elective percutaneous coronary intervention in Malaysia: a multicentre cross-sectional costing study. 2018; **7**: e014307.
41. Nourbala MH, Einollahi B, Kardavani B, et al. The cost of kidney transplantation in Iran. 2007; **39**: 927-9.
42. Odhiambo J, Ruhumuriza J, Nkurunziza T, et al. Health Facility Cost of Cesarean Delivery at a Rural District Hospital in Rwanda Using Time-Driven Activity-Based Costing. 2019; **23**: 613-22.
43. Ouakhzan B, Boukhalfa C. Application of the by the activity-based costing (ABC) method in a clinical department: example of the maternity unit of the Tiznit Provincial Hospital Center. 2020; **29**: 322-6.
44. Pejcic A, Radunovic A, Vulovic M. Analysis of inpatient costs in patients with knee osteoarthritis treated by implantation of total condylar knee endoprosthesis. *Vojnosanitetski Pregled* 2018; **75**(10): 989-97.
45. Portela MP, Neri ED, Fonteles MM, Garcia JH, Fernandes ME. The cost of liver transplantation at a university hospital of Brazil. 2010; **56**: 322-6.
46. Prinja S, Sharma Y, Dixit J, Thingnam SKS, Kumar R. Cost of Treatment of Valvular Heart Disease at a Tertiary Hospital in North India: Policy Implications. 2019; **3**: 391-402.
47. Qin X, Liu X, Huang J, et al. True cost of surgical aortic valve replacement and implications for price setting and diagnosis-related groups: evidence from a tertiary hospital in Eastern China. 2021; **10**: 697-708.
48. Rahmawati S, Graber MA, Hakimi M, Mukti AG, Bastian I, Rahman N. Cost comparison of emergency cesarean section in indonesia: The impact of australian model of diagnosis-related groups as a

payment system for patient care in hospitals. *Open Access Macedonian Journal of Medical Sciences* 2021; **9**: 216-23.
49. Rashidian A, Radinmanesh M, Afshari M, Arab M, Shahinfar Z. The comparison of the global surgery fees and the actual cost in two educational hospitals affiliated to Tehran University of Medical Sciences. *Evidence Based Health Policy, Management & Economics* 2018; **2**(4): 239-48.
50. Rizal AM, Aljunid SM, Normalina M, et al. Cost analysis of cataract surgery with intraocular lens implantation: a single blind randomised clinical trial comparing extracapsular cataract extraction and phacoemulsification. 2004; **58**: 380-6.
51. Ruhumuriza J, Odhiambo J, Riviello R, et al. Assessing the cost of laparotomy at a rural district hospital in Rwanda using time-driven activity-based costing. 2018; **2**: 25-33.
52. Saldarriaga EM, Carcamo CP, Babigumira JB, Garcia PJ. The Annual costs of treating genital warts in the Public Healthcare Sector in Peru. 2021; **21**: 1092.
53. Salgado Junior W, Pitanga KC, Santos JS, Sankarankutty AK, Silva Ode C, Jr., Ceneviva R. Costs of bariatric surgery in a teaching hospital and the financing provided by the Public Unified Health System. 2011; **25**: 201-5.
54. Salzedas-Netto AA, Gonzalez AM, Fagundes U, et al. Financial cost of the admissions for simultaneous pancreas-kidney transplant in a Brazilian hospital. 2015; **29**: 748-51.
55. Sangwan A, Prinja S, Aggarwal S, Jagnoor J, Bahuguna P, Ivers R. Cost of Trauma Care in Secondary- and Tertiary-Care Public Sector Hospitals in North India. 2018; **15**: 681-92.
56. Sarowar MG, Medin E, Gazi R, et al. Calculation of costs of pregnancy- and puerperium-related care: experience from a hospital in a low-income country. 2010; **28**: 264-72.
57. Sekeitto AR, Aden AA. Costing total hip arthroplasty in a South African state tertiary hospital. 2021; **111**: 250-4.
58. Sheriff S, Zawahrah HJ, Chang LV, et al. What is the Cost of Free Cleft Surgery in the Middle East? 2018; **42**: 1239-47.
59. Shetty SS, Moray KV, Chaurasia H, Joshi BN. Cost of managing atonic postpartum haemorrhage with uterine balloon tamponade devices in public health settings of Maharashtra, India: an economic microcosting study. 2021; **11**: e042389.
60. Singh MP, Prinja S, Rajsekar K, et al. Cost of Surgical Care at Public Sector District Hospitals in India: Implications for Universal Health Coverage and Publicly Financed Health Insurance Schemes. 2022; **6**: 745-56.
61. Singh MP, Chauhan AS, Rai B, Ghoshal S, Prinja S. Cost of Treatment for Cervical Cancer in India. 2021; **21**: 2639-46.
62. Soriano LG, Melo JRT. Costs of pediatric hydrocephalus treatment for the Brazilian public health system in the Northeast of Brazil. 2022; **38**: 2149-54.
63. Souza AB, Rodriguez S, Motta FLD, Brandao ABM, Marroni CA. The Cost of Adult Liver Transplantation in a Referral Center in Southern Brazil. 2019; **56**: 165-71.
64. Taazan B, Yamamoto E, Baatar B, et al. Estimating cost of hospitalization for childbirth at a tertiary hospital in Mongolia. 2021; **82**: 47-57.
65. Umo I, James K. The Direct Medical Cost of Acute Appendicitis Surgery in a Resource-Limited Setting of Papua New Guinea. 2022; **45**: 3558-64.
66. Van Heukelum M, Le Roux N, Jakoet S, Ferreira N. Financial burden of orthopaedic gunshot-related injury management at a major trauma centre. 2021; **110**: 882-6.
67. von Both C, Jahn A, Fleba S. Costing maternal health services in South Tanzania: a case study from Mtwara Urban District. 2008; **9**: 103-15.
68. Wang Y, Cui H, Zhang D, Zhang P. Hospitalisation cost analysis on hip fracture in China: a multicentre study among 73 tertiary hospitals. 2019; **8**: e019147.
69. Yang M, Hao J, Jian Z, Xiao YB, Zhou LX. Effects of Preoperative Risk Stratification on Direct In-hospital Costs for Chinese Patients with Coronary Artery Bypass Graft: A Single Center Analysis. 2019a; **38**: 1075-80.
70. Yang Y, Li PJ, Shuai T, et al. Cost analysis of oral and maxillofacial free flap reconstruction for patients at an institution in China. 2019b; **48**: 590-6.
71. Yereli AN. Activity-based costing and its application in a Turkish university hospital. 2009; **89**: 573-6, 9-91.
72. Younis M, Jabr S, Al-Khatib A, Forgione D, Hartmann M, Kisa A. A cost analysis of kidney replacement therapy options in Palestine. 2015; **52**.

73. Zhao W, Zhang L, Han S, et al. Cost analysis of living donor kidney transplantation in China: a single-center experience. 2012; **17**: 5-10.
74. Zhu L, Li J, Dong X, et al. Hospital costs and length of hospital stay for hepatectomy in patients with hepatocellular carcinoma:results of a prospective case series. 2012; **58**: 2052-7.
