# Supplementary material for: Costs of surgeries in low- and middle-income countries: a systematic literature review
Source: BMJ Glob Health. 2026 Sep 4;11(9):e020596. doi: 10.1136/bmjgh-2025-020596 (PMC13548284; doi:10.1136/bmjgh-2025-020596)
Supplement: Supplementary file 4 [file bmjgh-11-9-s004.pdf]

Supplemental material IV: Median and ranges of costs across procedure groups among the full costing studies.

S/N	Procedure group	Number of cost data points	Median	Maximum	Minimum
1	Genital warts treatment	3	46.11	59.22	37.09
2	Capsulotomy	1	94.78	94.78	94.78
3	Male circumcision	1	113.60	113.60	113.60
4	Cystoscopy	1	164.58	164.58	164.58
5	Electrosurgery	1	247.26	247.26	247.26
6	Surgical treatment	1	257.81	257.81	257.81
7	Cutaneaous swelling excision	1	313.19	313.19	313.19
8	Amputation	6	433.68	433.68	433.68
9	Injury surgical treatment	3	497.94	1,392.86	12.05
10	Lithotomy	1	509.77	861.29	239.80
11	Salpingectomy	2	515.45	689.77	341.14
12	Fasciectomy	1	527.48	527.48	527.48
13	Intestinal obstruction	1	538.61	538.61	538.61
14	Pterygium surgery	1	576.93	576.93	576.93
15	Hydrocelectomy	1	607.30	607.30	607.30
16	Glaucoma surgery	1	704.68	704.68	704.68
17	Septoplasty	1	745.89	745.89	745.89
18	Tympanoplasty	1	803.58	803.58	803.58
19	Adenoidectomy	24	911.97	1,510.75	313.19
20	Haemorrhoidectomy	2	935.45	935.45	935.45
21	Prostatectomy	1	980.91	980.91	980.91
22	Arthrodesis	8	1,083.81	1,083.81	1,083.81
23	Thyroidectomy	1	1,244.52	1,244.52	1,244.52
24	Adenectomy	10	1,330.81	1,330.81	1,330.81
25	Tonsillectomy	1	1,330.81	1,330.81	1,330.81
26	Hysterectomy	9	1,351.54	4,844.76	346.74
27	Caesarean section	3	1,381.59	4,785.41	1.54
28	Cranioplasty	10	1,400.04	1,521.57	1,278.50
29	Long bone fracture repair	1	1,511.75	2,316.73	706.76
30	Spine surgery	1	1,541.10	1,541.10	1,541.10
31	Mastectomy	1	1,557.72	1,557.72	1,557.72
32	Hernia repair	2	1,587.06	4,906.46	189.35
33	Myomectomy	2	1,594.47	1,889.37	1,334.56
34	Myelomeningocelectomy	1	1,718.36	1,718.36	1,718.36
35	Sinus surgery	1	2,125.67	4,489.62	684.08
36	Cholecystectomy	3	2,230.37	3,711.39	1,244.78
37	Craniotomy	1	2,689.33	6,388.44	1,098.56
38	Laparotomy	10	2,777.74	3,662.36	1,893.12
39	Cardiac defibrillator implantation	6	2,910.03	2,910.03	2,910.03
40	Hip fracture repair	2	4,119.12	7,383.92	964.30
41	Surgical debridement	1	4,412.29	4,412.29	4,412.29
42	Appendectomy	3	8,884.30	25,099.15	203.55

43	Cardiac valve repair	1	11,252.59	39,672.96	563.02
44	Cardiac revascularisation	5	13,729.64	19,161.14	10,046.79
45	Kidney transplant	5	19,174.31	19,174.31	19,174.31
46	Liver transplant	1	37,885.05	38,573.83	37,196.28
47	Heart transplant	2	84,237.01	167,393.60	644.41
48	Cataract surgery	3	84,337.52	618,098.20	247.26
49	Refractive surgery	1	164,948.50	164,948.50	164,948.50
