# Supplementary material for: Costs of surgeries in low- and middle-income countries: a systematic literature review
Source: BMJ Glob Health. 2026 Sep 4;11(9):e020596. doi: 10.1136/bmjgh-2025-020596 (PMC13548284; doi:10.1136/bmjgh-2025-020596)
Supplement: Supplementary file 5 [file bmjgh-11-9-s005.pdf]

Supplemental material 5: Median and ranges of costs across procedure groups among the partial costing studies

S/N	Procedure cluster	Number of cost data estimates	Median	Maximum	Minimum
1	Skin carcinoma treatment	1	411.93	411.93	411.93
2	Caesarean section	4	580.37	813.36	480.77
3	Hydrocephalus surgery	2	1,696.87	2,291.30	1,102.44
4	Orthopaedic tumour surgery	1	2,126.45	2,126.45	2,126.45
5	Cardiac revascularisation	3	2,586.38	22,566.91	2,495.15
6	Cataract surgery	4	2,705.20	3,473.51	1,439.91
7	Limb deformities surgery	1	3,267.96	3,267.96	3,267.96
8	Removal of orthopaedic hardware	1	3,376.48	3,376.48	3,376.48
9	Ankle & foot procedures	1	3,673.88	3,673.88	3,673.88
10	Elbow, shoulder and knee repairs	1	3,947.40	3,947.40	3,947.40
11	Laparotomy	1	4,417.63	4,417.63	4,417.63
12	Hepatectomy	4	4,776.90	5,759.00	4,469.00
13	Oral and maxillofacial reconstruction	3	5,732.90	5,921.19	1,046.36
14	Injury surgical treatment	2	6,513.88	8,744.19	4,283.58
15	Kidney transplant	3	7,057.02	20,988.00	6,573.55
16	Bariatric surgery	2	7,176.93	7,516.77	6,837.09
17	Catheter ablation	1	7,930.27	7,930.27	7,930.27
18	Hip fracture repair	8	8,450.59	18,333.76	352.35
19	Knee arthroplasty	5	11,093.14	21,084.43	6,536.30
20	Craniotomy	1	11,694.33	11,694.33	11,694.33
21	Amputation	1	12,481.47	12,481.47	12,481.47
22	Orthopaedic infection treatment	2	12,759.32	21,494.53	4,024.10
23	Heart transplant	1	15,983.77	15,983.77	15,983.77
24	Congenital heart surgery	2	17,778.44	18,879.31	16,677.56
25	Cardiac valve repair	1	29,982.13	29,982.13	29,982.13
26	Simultaneous pancreas-kidney transplantation	2	32,979.54	32,979.54	32,979.54
27	Simultaneous pancreas-kidney transplantation	1	41,632.74	41,632.74	41,632.74
28	Bile duct injury repair	1	49,965.34	49,965.34	49,965.34
29	Transplant surgery	1	76,326.43	76,326.43	76,326.43
