# Supplementary material for: Costs of surgeries in low- and middle-income countries: a systematic literature review
Source: BMJ Glob Health. 2026 Sep 4;11(9):e020596. doi: 10.1136/bmjgh-2025-020596 (PMC13548284; doi:10.1136/bmjgh-2025-020596)

Supplemental material 6: Rates of adherence to CHEERS checklist items among the included studies

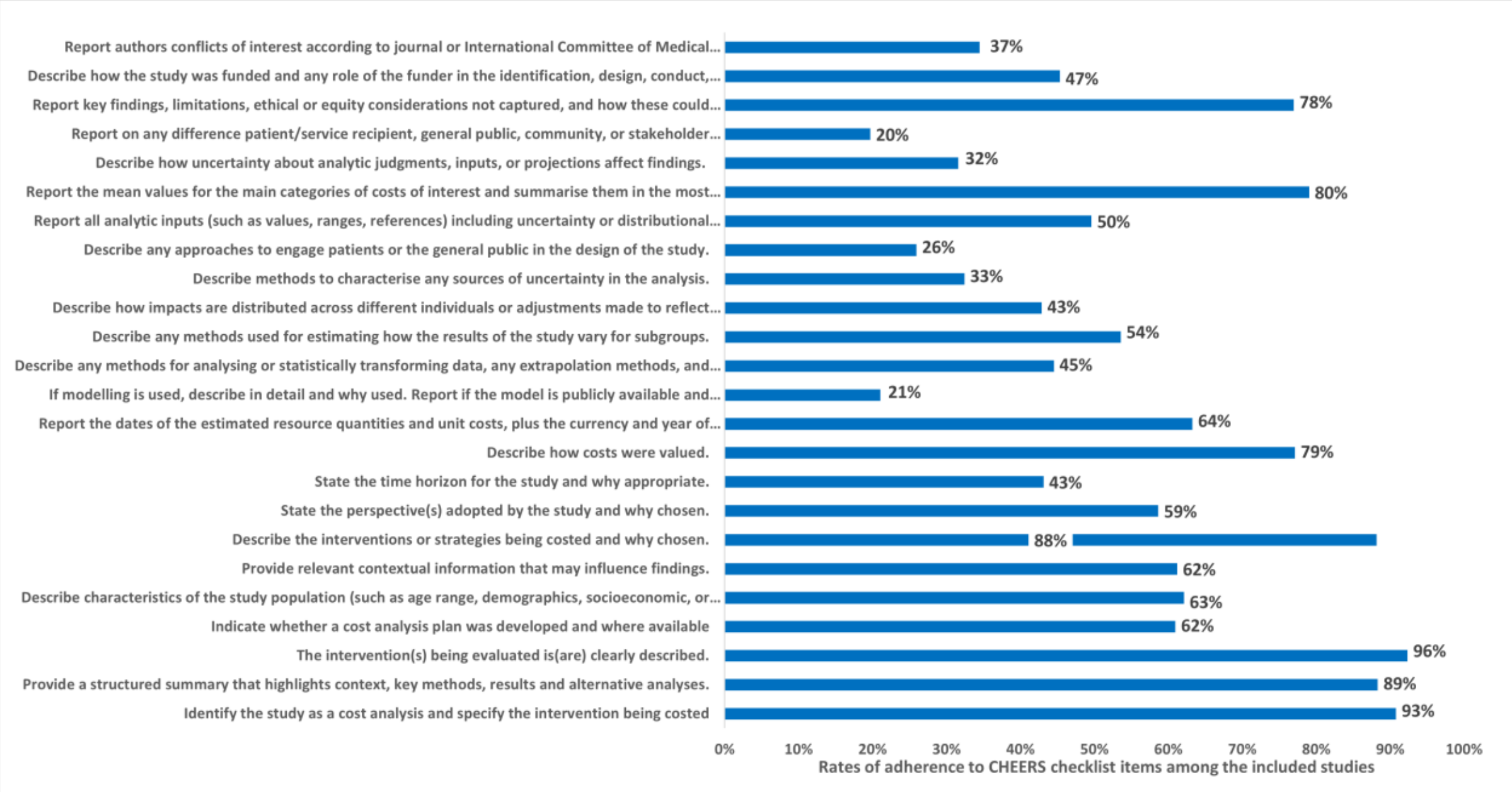

Supplement: Supplementary file 6 [file bmjgh-11-9-s006.pdf]
