# Supplementary material for: Costs of surgeries in low- and middle-income countries: a systematic literature review
Source: BMJ Glob Health. 2026 Sep 4;11(9):e020596. doi: 10.1136/bmjgh-2025-020596 (PMC13548284; doi:10.1136/bmjgh-2025-020596)
Supplement: Supplementary file 7 [file bmjgh-11-9-s007.pdf]

BMJ Global Health Author Reflexivity Statement

Adapted from Morton, B., Vercueil, A., Masekela, R., Heinz, E., Reimer, L., Saleh, S., Kalinga, C., Seekles, M., Biccard, B., Chakaya, J., Abimbola, S., Obasi, A. and Oriyo, N. (2022), Consensus statement on measures to promote equitable authorship in the publication of research from international partnerships. Anaesthesia, 77: 264-276. https://doi.org/10.1111/anae.15597

**Study conceptualisation**	
1. How does this study address local research and policy priorities?	An estimated 9 in 10 persons in LMICs lack access to safe, timely and affordable surgery. Results from this study could support planning and policies for strengthening surgical systems in the LMICs
2. How were local researchers involved in study design?	This study was a systematic review and therefore relied on existing literature materials. Nonetheless, collaborators were drawn from HICs and LMICs
**Research management**	
3. How has funding been used to support the local research team(s)?	The study was not funded
**Data acquisition and analysis**	
4. How are research staff who conducted data collection acknowledged?	The researchers who conducted the data collection are co-authors in the submission
5. How have members of the research partnership been provided with access to study data?	All the members the research team have free access to the data which were sourced from open literature
6. How were data used to develop analytical skills within the partnership?	All the research partners actively contributed to the data analysis in the research
**Data interpretation**	
7. How have research partners collaborated in interpreting study data?	Study results were shared with research partners and they all contributed in interpreting the findings
**Drafting and revising for intellectual content**	
8. How were research partners supported to develop writing skills?	All the research partners contributed in shaping the final manuscript after the lead author developed the first manuscript
9. How will research products be shared to address local needs?	Research findings will be shared with policymakers and other stakeholders in the forms of journal publication, policy briefs and conference presentations
**Authorship**	

10. How is the leadership, contribution and ownership of this work by LMIC researchers recognised within the authorship?	All the HIC and LMIC collaborators involved in the project are co-authors in the paper.
11. How have early career researchers across the partnership been included within the authorship team?	All the collaborators including senior and early career researchers on the project are co-authors in the paper.
12. How has gender balance been addressed within the authorship?	Seven authors are women while seven are men.
**Training**	
13. How has the project contributed to training of LMIC researchers?	The LMIC researchers were already experienced in research processes.
**Infrastructure**	
14. How has the project contributed to improvements in local infrastructure?	Not applicable (Study was not funded)
**Governance**	
15. What safeguarding procedures were used to protect local study participants and researchers?	Not applicable (Publicly available dataset was used)
